# Plastidial Expression of 3β-Hydroxysteroid Dehydrogenase and Progesterone 5β-Reductase Genes Confer Enhanced Salt Tolerance in Tobacco

**DOI:** 10.3390/ijms222111736

**Published:** 2021-10-29

**Authors:** Muhammad Sameeullah, Muhammet Yildirim, Noreen Aslam, Mehmet Cengiz Baloğlu, Buhara Yucesan, Andreas G. Lössl, Kiran Saba, Mohammad Tahir Waheed, Ekrem Gurel

**Affiliations:** 1 Department of Biology, Faculty of Science and Literature, Bolu Abant Izzet Baysal University, Bolu 14030, Turkey; sameepbg@gmail.com (M.S.); noreensamee206@gmail.com (N.A.); 2 Center for Innovative Food Technologies Development, Application and Research, Bolu Abant Izzet Baysal University, Bolu 14030, Turkey; 3 Department of Chemistry, Faculty of Science and Literature, Bolu Abant Izzet Baysal University, Bolu 14030, Turkey; muhammetyildirim@ibu.edu.tr; 4 Department of Genetics and Bioengineering, Faculty of Engineering and Architecture, Kastamonu University, Kastamonu 14030, Turkey; mcbaloglu@gmail.com; 5 Department of Seed Science and Technology, Faculty of Agriculture, Bolu Abant Izzet Baysal University, Bolu 14030, Turkey; ibuhara@yahoo.com; 6 Department of Applied Plant Sciences and Plant Biotechnology (DAPP), University of Natural Resources and Applied Life Sciences (BOKU), 1180 Vienna, Austria; andreas.loessl@boku.ac.at; 7 Department of Biochemistry, Faculty of Biological Sciences, Quaid-i-Azam University, Islamabad 45320, Pakistan; kiransaba128@gmail.com; 8 Department of Biochemistry, Faculty of Life Sciences, Shaheed Benazir Bhutto Women University, Peshawar 25000, Pakistan

**Keywords:** short chain dehydrogenase/reductase (SDR) genes, transplastomic plants, salt tolerance, 3β-HSD, NMR, sucrose, glutamate, glutamine, proline

## Abstract

The short-chain dehydrogenase/reductase (SDR) gene family is widely distributed in all kingdoms of life. The *SDR* genes, 3β-hydroxysteroid dehydrogenase (*3β-HSD*) and progesterone 5-β-reductases (*P5βR1*, *P5βR2*) play a crucial role in cardenolide biosynthesis pathway in the *Digitalis* species. However, their role in plant stress, especially in salinity stress management, remains unexplored. In the present study, transplastomic tobacco plants were developed by inserting the *3β-HSD*, *P5βR1* and *P5βR2* genes. The integration of transgenes in plastomes, copy number and transgene expression at transcript and protein level in transplastomic plants were confirmed by PCR, end-to-end PCR, qRT-PCR and Western blot analysis, respectively. Subcellular localization analysis showed that 3β-HSD and P5βR1 are cytoplasmic, and P5βR2 is tonoplast-localized. Transplastomic lines showed enhanced growth in terms of biomass and chlorophyll content compared to wild type (WT) under 300 mM salt stress. Under salt stress, transplastomic lines remained greener without negative impact on shoot or root growth compared to the WT. The salt-tolerant transplastomic lines exhibited enhanced levels of a series of metabolites (sucrose, glutamate, glutamine and proline) under control and NaCl stress. Furthermore, a lower Na^+^/K^+^ ratio in transplastomic lines was also observed. The salt tolerance, mediated by plastidial expression of the *3β-HSD*, *P5βR1* and *P5βR2* genes, could be due to the involvement in the upregulation of nitrogen assimilation, osmolytes as well as lower Na^+^/K^+^ ratio. Taken together, the plastid-based expression of the *SDR* genes leading to enhanced salt tolerance, which opens a window for developing saline-tolerant plants via plastid genetic engineering.

## 1. Introduction

*Digitalis* species, known as foxglove, are famous for the production of secondary metabolites known as cardiac glycosides, which have pharmaceutical importance in cardiac arrest and also possess anti-cancer activities [1,2]. The genes 3β-hydroxysteroid dehydrogenase (*3β-HSD*) and progesterone 5-β-reductases (*P5βR1*, *P5βR2*) are among the important key step genes in the pathway of biosynthesis of cardenolide in *Digitalis* species [3,4,5]. During the last two decades, extensive studies have been conducted on the cardiac glycosides, pathways and substrates for the glycosides and recombinant protein production of the *3β-HSD*, *P5βR1* and *P5βR2* using a bacterial heterologous expression system [4,5,6,7,8]. The cardenolides biosynthesis is also known to be triggered by the stresses like heat, cold, wound, submergence in water, hydrogen peroxide (H_2_O_2_), a precursor of ethylene biosynthesis: 1-aminocyclopropane-1-carboxylic acid (ACC), as well as drought and nutrient deficiency in soil [8]. To understand the cardenolide biosynthesis, most of the studies have employed tissue culture methods [2,8,9,10]. The genes *3β-HSD* [11], *P5βR1* [6] and *P5βR2* [8] were isolated from *Digitalis lanata*. The recombinant proteins were able to digest the respective substrates [5,6,8]. Although extensive studies of enzymatic reactions, crystal structure and substrate specificity have been conducted, until now, the functional analysis of these genes in transgenic plant studies has not been a focus of study.

Few reports are available about the genetic transformation of *Digitalis* species and marker genes were transformed to establish the *Agrobacterium*-mediated transformation for nuclear transformation. For the first time, Saito et al. [12,13] established *A*. *tumefaciens*- and *A. rhizogenes*-mediated transformation of *D. purpurea* using GUS or an antibiotic marker gene. Later, Lehmann et al. [14] reported the first transgenic *D. lanata* plants by *A. tumefaciens* using protoplast cells. In short, the genetic transformation reported till date for the *Digitalis* species has been done to optimize the transformation system [15,16,17,18]. Sales et al. [19] studied the cardenolide pathway by using the 3-hydroxy-3-methylglutaryl coenzyme A reductase (*HMG1*) gene from Arabidopsis by transforming into *D. minor*. Cardenolide 16′-O-glucohydrolase I encoded by the Cardenolide 16′-O-glucohydrolase I (*CGH I*) gene from *D. lanata* EHRH was transformed into the roots of *Cucumis sativus* L. to make an effort to produce cardenolides [20]. However, the level of cardenolides was lower than in the leaves of wild-type *D. lanata* because the site of biosynthesis of cardenolides is mainly chlorophyllous organs and not the non-chlorophyllous [21]. To date, the *3β-HSD*, *P5βR1* and *P5βR2* genes have not yet been transformed into either chloroplast or nuclear genome for their functional analysis under salt stress.

Tobacco has been commonly used as a model plant for plastid transformation since it has a short life cycle, is easy to grow via tissue culture, and can produce huge biomass and seeds. One of the main advantages of chloroplast transformation over nucleus transformation is very high expression, due to high copy number of transgenes (up to 10,000 copies) in a single tobacco leaf cell [22]. Plastid transformation is based on homologous recombination where the vector′s flanking regions contain sequences of the plastid genome ensuring correct insertion within the plastid genome via homologous recombination. This technique also minimizes the chances of off-target genes silencing [23]. The plastome is strictly maternally inherited in most species of agricultural interest [24]. Therefore, the risks of transgene dispersal via pollen are diminished.

Soil salinity is a serious abiotic stress which restricts crop productivity severely. More than 800 million hectares of land is salt-affected in the world, and this amount is still rising. Salinity has become a growing threat to sustainability of agriculture worldwide [25,26,27]. One effective approach to improve the productivity of salt-stressed soils is to breed salt-tolerant crop cultivars [28]. Another alternative is the use of transformation technologies by inserting genes that mediate salt tolerance in plants.

Since, cardenolide biosynthesis is reported to be induced due to the abiotic stress factors [3,29], the aim of this study was to explore the potential functional role of selected *SDR* genes, *3β-HSD*, *P5βR1* and *P5βR2* from *D. ferruginea* subsp. *ferruginea*, under salinity stress by expressing these genes in plastomes of *Nicotiana tabacum*. The expression of these genes led to enhanced salt tolerance in the developed transplastomic tobacco plants under high salt stress. The transplastomic plants remained green, retained high chlorophyll contents and showed high biomass under salinity stress.

## 2. Results

### 2.1. Generation of Transplastomic Plants Expressing 3β-HSD, P5βr1 and P5βr2 Genes

#### 2.1.1. Plastid Transformation Vectors and Development of Transplastomic Plants

**Figure 1:**
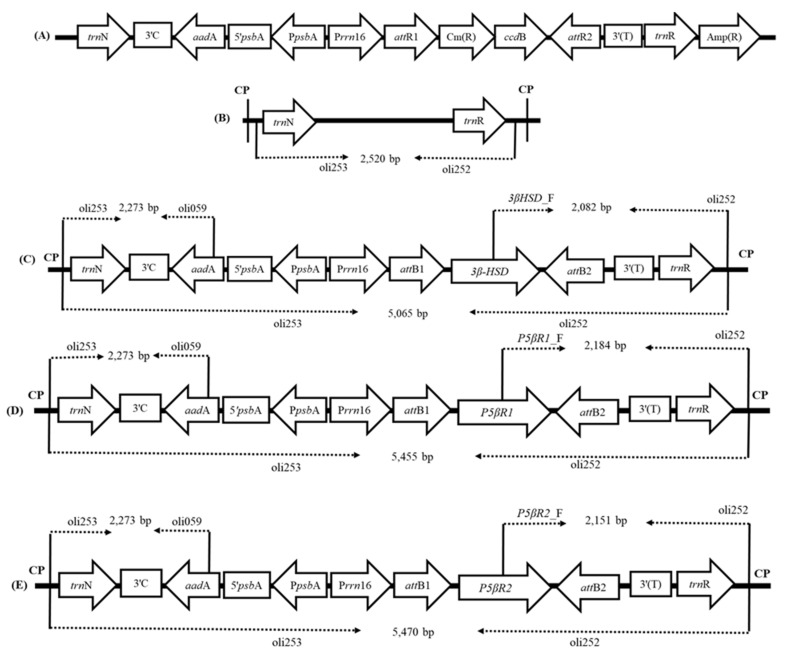
Cloning steps and vector construction. (**A**) Schematic representation of pDEST-PN-T. The Gateway^®^ compatible destination vector used for cloning contains the chloramphenicol resistance gene (Cm(R)) and the control of cell death gene (*ccdB*) flanked by the Gateway^®^ recombination sites *att*R1 and *att*R2. Amp(R): ampicillin resistance gene; (**B**) schematic representation of the targeting region in the wild-type tobacco plastid genome. The transgene expression cassette was targeted for insertion into the plastid genome (CP) in the intergenic spacer region between *trn*N and *trn*R. Expected fragment of end-to-end PCR for wild type using primer pair oli252 and oli253 was 2520 bp; (**C**) final transformation vector pEXP-PN-T-3β-HSD: shows expression cassette inserted within tobacco chloroplast genome; (**D**) final transformation vector pEXP-PN-T-P5βR1, showing expression cassette inserted within tobacco chloroplast genome, and (**E**) final transformation vector pEXP-PN-T-P5βR2 which shows expression cassette inserted within tobacco chloroplast genome. All positions of primers are given along with their expected fragment sizes. *Prrn*16: constitutive P*rrn*PEP+NEP promoter; *3β-HSD*: 3β-hydroxysteroid dehydrogenase; *P5βR1*: progesterone 5β-reductase 1; *P5βR2*: progesterone 5β-reductase 2; PpsbA: promoter psbA; 3′T: 3′UTR of tobacco *rbc*L; 3′C: 3′UTR of *Chlamydomonas reinhardtii rbc*L; 5′psbA: 5′UTR of *psbA* gene; attR1/R2/B1/B2: Gateway^®^ recombination sites.

For plastid transformation, pEXP-PN-T-3β-HSD, pEXP-PN-T-P5βR1 and pEXP-PN-T-P5βR2 were constructed for the transformation of the *3β-HSD*, *P5βR1* and *P5βR2* separately into tobacco plastid genome. The final plastid expression vectors consisted of *3β-HSD*, *P5βR1* and *P5βR2* genes under control of constitutive *Prrn*PEP+NEP promoter. Each vector also contained an *aadA* gene cassette conferring resistance to antibiotics spectinomycin and streptomycin for selection of transplastomic plants. The expression of *aadA* gene was controlled by promoter P*psb*A and T*rbc*L terminator. The flanking sequences for targeted homologous recombination of the expression cassette into the plastid genome of *N*. *tabacum* were *trn*N and *trn*R, located in the inverted repeat (IR) region. Complete scheme of vector construction is given in Figure 1. The transplastomic plants were generated by gene gun-mediated DNA delivery and the transformants were selected on RMOP media containing 500 mg/L spectinomycin [30].

#### 2.1.2. Confirmation of Transgene Integration

**Figure 2:**
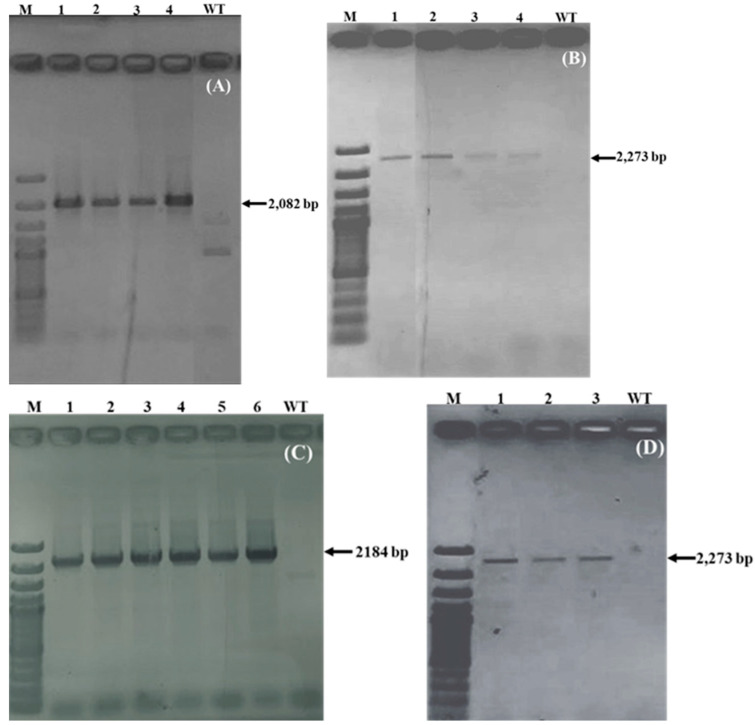

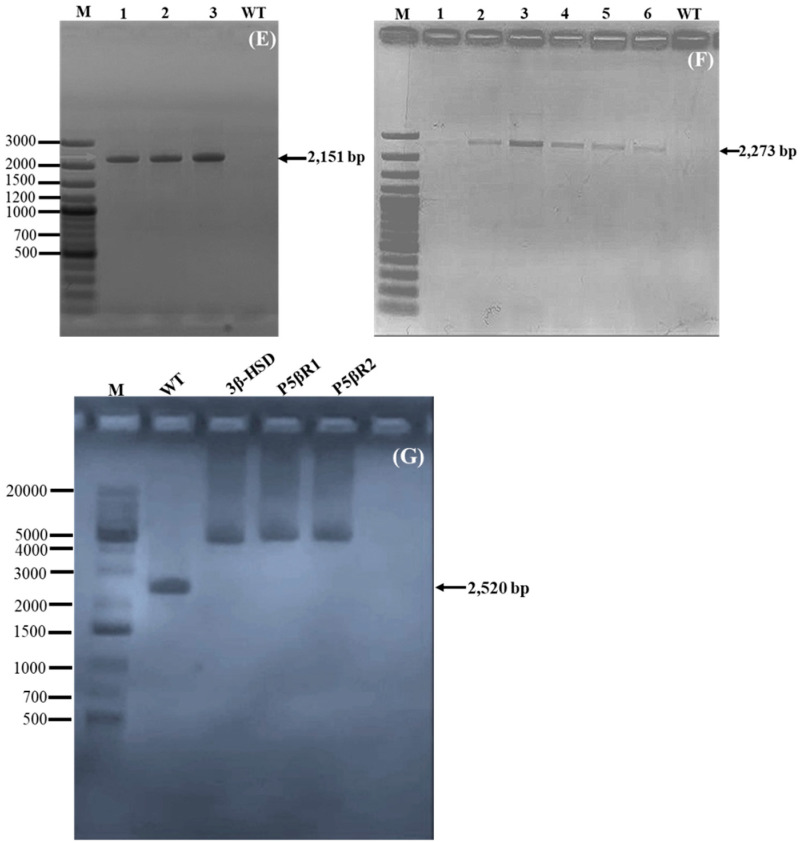
Transgene and homoplasmy confirmation in transplastomic tobacco plants by PCR. (**A**) Confirmation of correct insertion of the *3β-HSD* gene within the plastome on *trn*R side with 2082 bp amplified product, (**B**) Confirmation of correct integration of the *aadA* gene within the transplastomic plants containing *3β-HSD* gene, an amplicon of 2273 bp was obtained, (**C**) Confirmation of correct insertion of the *P5βR1* gene within the plastome on *trn*R side with 2184 bp amplified product, (**D**) Confirmation of correct integration of *aadA* gene within the transplastomic plants containing the *P5βR1* gene, an amplicon of 2273 bp was obtained, (**E**) Confirmation of correct insertion of *P5βR2* gene within the plastome on *trn*R side with 2151 bp amplified product and (**F**) Confirmation of correct integration of the *aadA* gene within the transplastomic plants containing the *P5βR2* gene, an amplicon of 2273 bp was obtained. M: Marker 100 bp plus: Lanes 1,2,3,4,5,6: independent transgenic lines of the *3β-HSD*, *P5βR1* and *P5βR2*. WT: wild-type tobacco as negative control. (**G**) End-to-end PCR for the confirmation of homoplasmy in transplastomic tobacco plants. The expected product length 5065 bp for the gene *3β-HSD* was observed on gel. Amplified fragments of 5455 bp and 5470 bp in sizes were obtained in transplastomic plants containing *P5βR1* and *P5βR1* genes, respectively. A 2520 bp fragment was obtained in WT tobacco plants. No band of WT DNA was detected in transplastomic plants showing that transplastomic tobacco plants contained only transformed state. M: marker; WT: wild-type tobacco plant; *3β-HSD*, *P5βR1*, *P5βR1*: transplastomic plants containing the *3β-HSD*, *P5βR1* and *P5βR1* genes, respectively. M: Marker 1 kb plus.

PCR was performed to verify the correct integration of the *3β-HSD*, *P5βR1* and *P5βR2* genes within the plastid genome of tobacco. Confirmation of the presence of *3β-HSD* gene was done by PCR amplification by using primers 3βHSD_F (located within the *3β-HSD* gene) and oli252 (positioned within the plastome outside the right flank (*trn*R)). An amplicon size of 2082 bp was obtained as expected (Figure 2A–F). To confirm the presence of the *P5βR1* gene within the plastid genome, we used primers P5βR1_F (positioned within the *P5βR1* gene) and oli252 (positioned within the plastome outside the right flank (*trn*R)). An amplicon size of 284 bp was obtained as expected. Confirmation of the presence of *P5βR2* gene was done by PCR amplification by using primers P5βR2_F (located within the *P5βR2* gene) and oli252 (positioned within the plastome outside the right flank (*trn*R)). An amplicon size of 2151 bp was obtained. For confirmation of the correct insertion of the *aadA* gene within the transplastomic plants containing the *3β-HSD*, *P5βR1* and *P5βR2* genes, a set of primer oli253 (located within the plastid genome outside the left flank (*trn*N)) and oli059 (located within the *aadA* gene) were used. An amplicon of 2273 bp was obtained in transplastomic plants containing *3β-HSD*, *P5βR1* and *P5βR2* genes. Figure 2 shows all amplified product as obtained on agarose gel.

#### 2.1.3. Expression of Transgene RT-qRT-PCR

**Figure 3:**
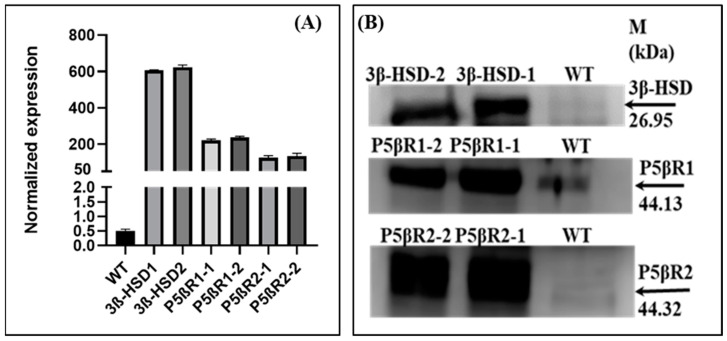
(**A**)Transgene expression of the *3β-HSD*, *P5βR1* and *P5βR2* by real time qRT-PCR in independent transplastomic plant lines 3βHSD-1, 3βHSD-2, P5βR1-1, P5βR1-2, P5βR2-1 and P5βR2-2. Real-time reverse transcription-polymerase chain reaction (RT-qRT-PCR) was performed, using gene specific primer set for the *3β-HSD*, *P5βR1* and *P5βR2*. Relative expression levels were normalized against the values of the *Actin9* transcripts in WT and the transplastomic lines. Each value represents the mean ± standard error (SE) of three samples from three independent experiments. (**B**) Western blot analysis of 3β-HSD, P5βR1 and P5βR2 in transplastomic lines 3β-HSD-1, 3β-HSD-2, P5βR1-1, P5βR1-2, P5βR2-1, P5βR2-2 and WT tobacco. The molecular weights of the protein were 26.95 kDa, 44.13 kDa and 44.32 kDa for 3β-HSD, P5βR1 and P5βR2, respectively. M: Marker. WT: Wild type. 3β-HSD-1, 3β-HSD-2: Two independently generated lines of 3β-HSD. P5βR1-1, P5βR1-2: Two independently generated lines of P5βR1. P5βR2-1, P5βR2-2: Two independently generated lines of P5βR2.

Transgene expression of the *3β-HSD*, *P5βR1* and *P5βR2* was determined by isolating total RNA from WT and the independent transplastomic lines 3β-HSD-1, 3β-HSD-2, P5βR1-1, P5βR1-2, P5βR2-1 and P5βR2-2. cDNA was used for real time qRT-PCR, which was synthesized using genomic DNA free RNA. The transgene expression was very high in all independent lines of the *3β-HSD*, *P5βR1* and *P5βR2* tobacco transplastomic plants. The transplastomic lines of the *3β-HSD* exhibited the highest transgene expression while some degree of expression was also detected in WT (Figure 3A). These results show that the transgenes *3β-HSD*, *P5βR1* and *P5βR2* were efficiently expressed in the transplastomic tobacco plants.

#### 2.1.4. End-to-End PCR

End-to-end PCR can indicate any untransformed plastid genomes. PCR was performed by using sense primer oli253 (located within the chloroplast genome outside of the *trn*N of the expression cassette) and anti-sense primer oli252 (positioned within the chloroplast genome outside of the *trn*R). An amplicon size of 2520 bp was obtained for wild-type untransformed plants. For transplastomic plants containing *3β-HSD* gene, an amplicon of 5065 bp was obtained. In transplastomic plants containing *P5βR1* gene, we obtained the fragment size of 5455 bp. In transformed plants containing *P5βR2* gene, the fragment size obtained was 5470 bp. The absence of 2520 bp band in transplastomic lines showed that no wild-type copy of plastid genome was left and the developed transplastomic plants were homoplasmic, containing all copies of plastomes transformed. The expected sizes of the fragments and positions of primers are shown in Figure 1. Fragments obtained after PCR are shown on agarose gel in Figure 2G.

### 2.2. Western Blot

Western blot was performed to detect the protein in transplastomic lines using soluble protein fraction. Crude antisera (anti-3β-HSD, anti-P5βR1, anti-P5βR2) were used to detect the expressed proteins. Western blot analysis showed the protein bands of 26.95 kDa, 44.13 kDa and 44.32 kDa of 3β-HSD, P5βR1 and P5βR2, respectively (Figure 3B) in the transplastomic tobacco plants. However, a faint band was also observed in case of WT plants showing some degree of expression. The intensity of band in WT was far less as compared to transplastomic plants. These results showed that functional protein was synthesized in independent lines of the transplastomic plants transformed with the genes *3β-HSD*, *P5βR1* and *P5βR2*.

### 2.3. Subcellular Localization of 3β-HSD, P5βR1 and P5βR2

*Agrobacterium*-mediated transformation of pGWB5-35S::3β-HSD-GFP, pGWB5-35S::P5βR1-GFP, pGWB5-35S::P5βR2-GFP and pGWB5-35S::GFP under the control of a 35S promoter was performed in onion cells. Expression of 3β-HSD and P5βR1 was distributed thoroughly as tiny vesicles in cytoplasm possibly due to their localization on ribosomes, in mitochondria (3β-HSD) or endoplasmic reticulum (P5βR1) [31]. On the other hand, the expression of P5βR2 was found to be tonoplast-localized (Supplementary Figure S1). In case of GFP control, the GFP expression was observed in the nucleus, cytoplasm and cell membrane. These results show that 3β-HSD and P5βR1 proteins have affinity to the cytoplasm, while P5βR2 is a vacuolar membrane-localized protein.

### 2.4. Effect of Salinity on Plant Growth and Development

**Figure 4:**
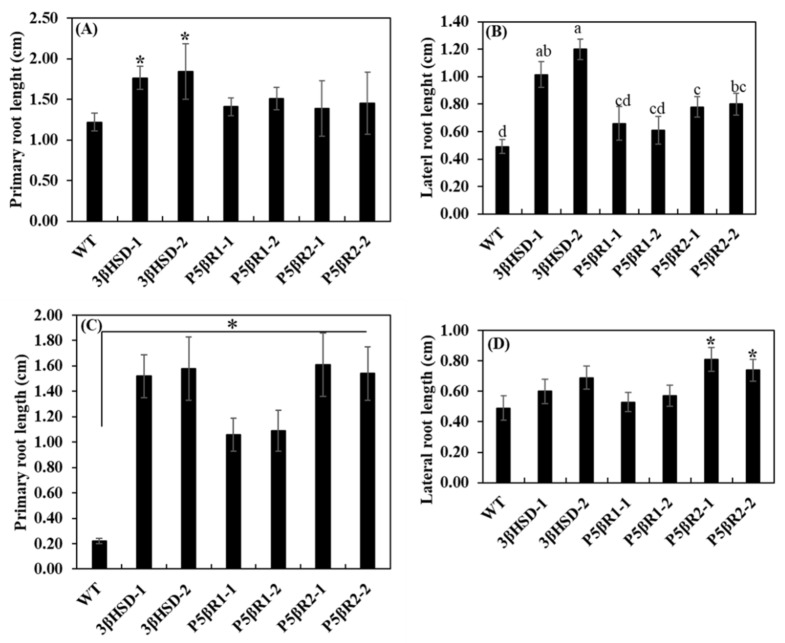
Primary and lateral root lengths of WT and transplastomic lines of the *3β-HSD*, *P5βR1* and *P5βR2* genes under control (0 mM NaCl) and 300 mM of NaCl stress on MS agar media over one month. (**A**) Primary root lengths of WT and two independent transgenic lines each of the *3β-HSD*, *P5βR1*, and *P5βR2* genes under the control treatment; (**B**) lateral root lengths of the seedlings under the control treatment; (**C**) primary root lengths of the seedlings under 300 mM NaCl stress; (**D**) lateral root lengths of salt-stressed (300 mM NaCl) seedlings. Asterisks or different letters show significant differences at *p* < 0.05 (WT, *n* = 3, transplastomic lines, *n* = 8 for each repeat of each line) among the WT and the lines in each figure. Data shows average of three repeats and error bars represent ± SE.

The plant growth and development were assessed under control and salt stress treatments based on the primary and lateral root length as well as fresh biomass of WT and independently generated transplastomic lines of the *3β-HSD*, *P5βR1* and *P5βR2*. In control treatments, transplastomic lines exhibited longer primary and lateral root lengths than WT (Figure 4A,B). These results suggest that transplastomic expression of the *3β-HSD* resulted in the enhancement of primary and lateral root lengths in tobacco plants. Under the salt stress at various concentrations of 50 mM (Supplementary Figure S2A,B), 200 mM (Supplementary Figure S2C,D) and 300 mM (Figure 4C,D), primary and lateral root lengths of transplastomic lines were longer than WT tobacco plants. These results suggest that the transplastomic expression of the *3β-HSD*, *P5βR1* and *P5βR2* attributed to the tolerance of salt stress in transplastomic tobacco plants (Figure 4C).

**Figure 5:**
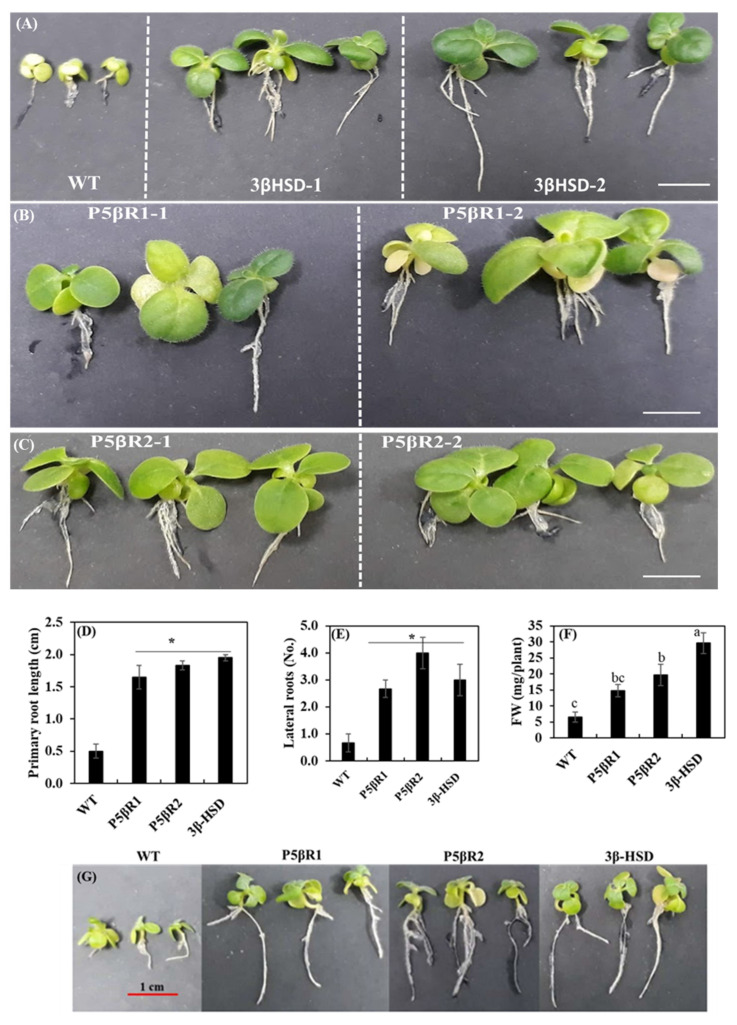
Phenotype of WT and the independently generated transplastomic lines of the *3β-HSD*, *P5βR1* and *P5βR2* genes (3βHSD-1, 3βHSD-2, P5βR1-1, P5βR1-2, P5βR2-1 and P5βR2-2) under salt and mannitol stresses. Impact of salt stress on the phenotype of WT and transplastomic lines when exposed to 300 mM NaCl on MS media. Photographs were taken after 30 days of culture. Independent transplastomic lines of *3β-HSD* (**A**); *P5βR1* (**B**); and *P5βR2* (**C**) exhibited bigger green leaves and longer roots than WT. The osmotic stress was applied to root excised seven-day-old seedlings at 400 mM mannitol concentration for the period of 25 days on MS media. After 25 days, the phenotypic data of WT and transplastomic seedlings for root length, lateral root number and fresh weight of shoots was measured. WT and transplastomic seedlings (*n* = 15). (**D**) The significant difference for root length among WT and transplastomic seedlings is indicated by an asterisk. (**E**) The significance difference for lateral root number among WT and transplastomic seedlings is indicated by an asterisk. (**F**) The significance differences for shoot FW among WT and transplastomic seedlings are indicated by letters of thrice-replicated data. Different letters show significant differences at *p* < 0.05 (*n* = 5 each repeat of each line) for three repeats. (**G**) Impact of osmotic stress on the phenotype of WT and transplastomic lines when exposed to 400 mM mannitol using MS media. Photographs were taken after 25 d of culture. Scale bar length is 1 cm.

Fresh weights of plants were also determined in control and salt stress treatments of WT and transplastomic lines (3β-HSD-1, 3β-HSD-2 P5βR1-1, P5βR1-2, P5βR2-1 and P5βR2-2) expressing *3β-HSD*, *P5βR1* and *P5βR2* genes, respectively. Under control treatment, transplastomic lines showed higher plant fresh weight (FW), shoot FW and root FW than WT tobacco plants (Supplementary Figure S3A). At a low concentration of NaCl (50 mM) treatment, the difference among fresh biomass of WT and transplastomic lines was very minute (Supplementary Figure S3B). However, at higher concentrations (200 mM in Supplementary Figure S4C and 300 mM in Supplementary Figure S3D) of NaCl, fresh biomass (plant, shoot and root FW) was remarkably decreased in WT as compared to transplastomic lines. At the highest concentration (300 mM) of NaCl, plant FW, shoot FW and root FW were maintained in the transplastomic lines but severely reduced in WT (Supplementary Figure S3D). Further, total chlorophyll content in WT and the transplastomic lines grown under highest applied salt stress of 300 mM NaCl was measured. Transplastomic lines retained considerable chlorophyll content while deteriorated and lower chlorophyll content was observed in WT (Supplementary Figure S4), suggesting that the transplastomic lines enhanced protection of the photosynthetic machinery. The phenotype of transplastomic lines was maintained under 300 mM NaCl than WT (Figure 5A) with retarded growth. Further experiment was conducted to determine the effect of osmotic stress on the WT and transplastomic lines. Four hundred mM of mannitol stress for 25 days imposed drastic effect on primary root length (5D), lateral root length (5E), FW (5F) and phenotype (5G) of WT than transplastomic lines, which exhibited significantly higher growth in terms of primary, lateral root lengths and FW. These results further confirm that the transplastomic lines also cope with osmotic stress. These results indicated that transplastomic expression of the *3β-HSD*, *P5βR1* and *P5βR2* genes maintained primary and lateral root lengths, fresh biomass, and improved plant growth and development under the stresses by ameliorating the toxic effects of NaCl or mannitol stress as evident from Figure 5.

### 2.5. Metabolite Analysis of the Plants Grown under Salt Stress

**Figure 6:**
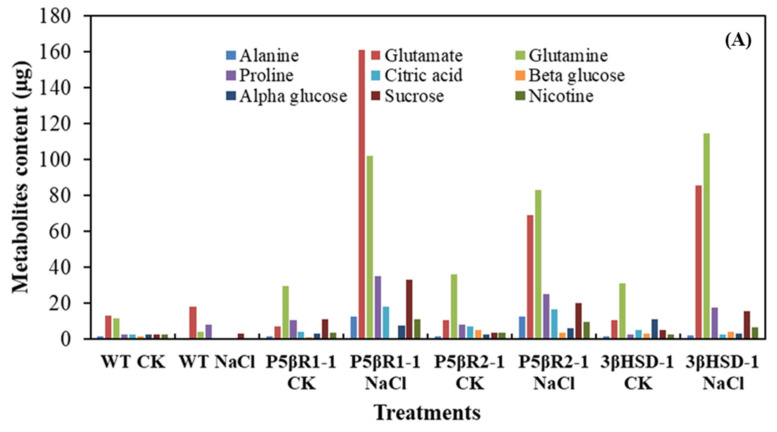

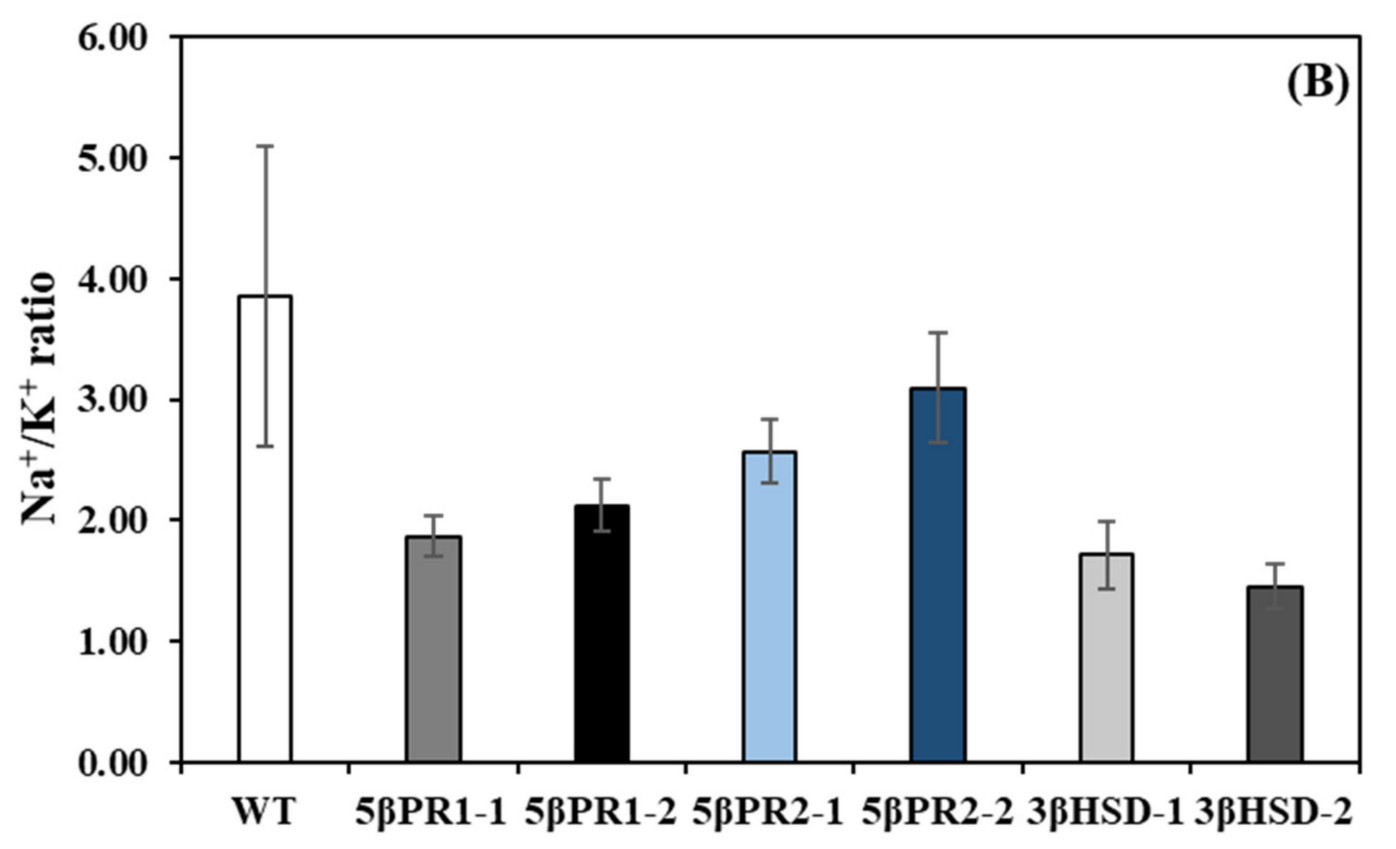
NMR-based metabolites in WT and transplastomic (P5βR1, P5βR2, 3β-HSD) lines in control (CK) and under salt stress (300 mM NaCl). (**A**) The three seedlings of each line or WT were used for the metabolites analysis. (**B**) The Na^+^/K^+^ ratio in WT and the independently generated transplastomic lines of the *3β-HSD*, *P5βR1* and *P5βR2* genes (P5βR1-1, P5βR1-2, P5βR2-1, P5βR2-2, 3β-HSD-1 and 3β-HSD-2) grown under NaCl (300 mM) stress. Data shows average of three repeats and error bars represent ± SE.

NMR-based determination of metabolites was carried out in one-month old WT and transplastomic lines grown under 300 mM salt stress. Metabolites were at the lowest level under control treatment (no salt stress) both in WT and the transplastomic (3β-HSD-1, P5βR1-1 and P5βR2-1) lines. However, a small increase in glutamine level was observed in transplastomic lines under control treatment (Figure 6A). At 300 mM NaCl concentration, glutamate and glutamine levels were enhanced dramatically in transplastomic lines in comparison with WT. An increase was also observed in proline and sucrose content. In comparison to WT under salt stress or in control, a minute increment in glutamate and proline content was observed in WT due to salt stress (Figure 6A). The enhanced levels of glutamate, glutamine, proline and sucrose might contribute to salt tolerance in transplastomic plants by increasing the osmotic pressure within the plant cell.

The Na*^+^*/K*^+^* ratio was calculated in WT and transplastomic lines of the *P5βR1*, *P5βR2* and *3β-HSD*. The ratio of Na*^+^*/K*^+^* was found higher (3.85 mg/g DW) than all of the transplastomic plants. The P5βR1-1 and P5βR1-2 lines of the *P5βR1* showed ratio of Na*^+^*/K*^+^* equivalent to 1.87 and 2.13 mg/g DW, respectively. This ratio was calculated to 2.57 mg/g and 3.09 mg/g DW for transplastomic lines P5βR2-1 and P5βR2-2 of the *P5βR2*, respectively. The independent transplastomic lines 3β-HSD-1 and 3β-HSD-2 of the *3β-HSD* exhibited 1.72 mg/g and 1.45 mg/g DW, respectively (Figure 6B). These results suggested that the transplastomic expression of the *P5βR1*, *P5βR2* and *3β-HSD* exhibited magnificent resistance to salt stress by accumulating less Na*^+^* concentration as compared to WT.

### 2.6. Transplastomic Expression of the P5βr1, P5βr2 and 3β-HSD Decreases Na^+^/K^+^ Ratio under Salt Stress

Levels of Na^+^ and K^+^ were determined in the WT and independently generated *P5βR1*, *P5βR2* and *3β-HSD* expressing transplastomic lines grown under salt stress of 300 mM NaCl over the period of one month. Na^+^ concentration in the leaves of wild-type tobacco plant was 14.08 mg/g dry weight (DW). In comparison, the transplastomic lines of the *P5βR1* showed highest levels of Na^+^ up to 63.87 mg/g and 62.66 mg/g DW for P5βR1-1 and P5βR1-2, respectively. The lines P5βR2-1 and P5βR2-2 of the *P5βR2*-transformed plants contained 42.12 and 37.97 mg/g DW of Na^+^, respectively. The transplastomic lines 3β-HSD-1 and 3β-HSD-2 of the *3β-HSD* exhibited 42.20 and 18.97 mg/g DW of Na^+^ (Supplementary Figure S5). The concentration of K^+^ in WT was 4.28 mg/g of DW. The level of K^+^ in transplastomic lines P5βR1-1 and P5βR1-2 of P5βR1 was 35.11 mg/g and 29.92 mg/g DW, respectively. The lines P5βR2-1 and P5βR2-2 of P5βR2 accumulated 16.76 and 12.95 mg/g DW, respectively. The transplastomic lines 3β-HSD-1 and 3β-HSD-2 of the 3β-HSD showed 14.82 mg/g and 13.63 mg/g DW, respectively (Supplementary Figure S6).

## 3. Discussion

### 3.1. Contribution of 3β-HSD, P5βR1 and P5βR2 Genes towards Plant Growth and Development

The substrate for 3β-HSD is pregnenolone which is converted into progesterone due to enzymatic reaction. However, there are several substrates for the enzymes P5βR1 and P5βR2, such as 1,4-enones, such as 2-cyclohexen-1-one, methyl vinyl ketone or citral are also accepted [32] but progesterone exhibits more specificity as a substrate [3]. Secondary metabolites (cardenolides and progesterone) biosynthesis is also triggered due to abiotic (salt, wound, heat, cold) [3] or biotic stress factors (pathogen or insect herbivory) [33]. Therefore, keeping in consideration such inducible factors for the regulation of the *3β-HSD*, *P5βR1* and *P5βR2* genes we hypothesized that perhaps plastidial expression of these genes would confer salt tolerance in the present research. Further, our hypothesis was strengthened as some orthologous gene from SDR family were induced due to salt stress in bacteria [34] or plant [3] and the recent findings further strengthening our hypothesis [35,36].

In the present study, we expressed the *3β-HSD*, *P5βR1* and *P5βR2* genes encoding short chain dehydrogenase reductase from *D. ferruginea* subsp. *ferruginea* [4,37] and investigated their role in plant growth under normal and NaCl-stressed conditions. For the functional genomics study, we developed chloroplast transformed tobacco plants to express the *3β-HSD*, *P5βR1* and *P5βR2* genes. By immunoblot analysis, 3β-HSD, P5βR1 and P5βR2 were recognized as a 26.95, 44.13 and 44.32-kD proteins, respectively. PCR analysis of transplastomic plants confirmed the integration of transgenes into chloroplast genome of tobacco. Further, end-to-end PCR confirmed the presence of only transformed plastomes in transplastomic plants. Real-time qPCR analysis of the independent transplastomic lines 3β-HSD-1, 3β-HSD-2, P5βR1-1, P5βR1-2, P5βR2-1 and P5βR2-2 revealed an efficient transgene expression compared to a negligible expression in WT. The over-expression of the *3β-HSD*, *P5βR1* and *P5βR2* genes resulted in better growth rates in terms of enhanced primary and lateral roots as well as fresh shoot and root weight compared to WT under normal growth conditions.

It is well-established that Na^+^ in low concentration (the concentration which is not harmful to plants) also stimulates the growth and development of the plants and act as a beneficial nutrient [38]. We could observe that under 50 mM or 200 mM NaCl treatments, the growth of tobacco seedlings (WT and transplastomic lines) was higher (more prominent in transplastomic lines) than the control treatment (Supplementary Figures S2 and S3). It is speculated that the difference among control (0 mM NaCl) and 200 mM NaCl treatments could possibly be due to beneficial effects of Na on the seedlings’ growth. In our findings, to support the better growth of transplastomic lines under control treatment, Na^+^ concentration was higher in transplastomic than WT (Supplementary Figure S5). Similarly, the K^+^ concentration was also found remarkable high in the transplastomic plants than WT under control (Supplementary Figure S6), which is one of the most important macronutrients and plays critical role in plant development [39]. *AtSDR1* (an orthologue of the *3β-HSD*, *P5βR1* and *P5βR2*), which is also known as Glucose Insensitive1 (*GIN1*) and Abscisic Acid Deficient2 (*ABA2*) are reported to be involved in abscisic acid biosynthesis, which also modulates the plant growth and development. It is also reported that the mutant of the *AtSDR1* governed the poor and stunted growth of Arabidopsis plants [40]. Another study demonstrated that overexpression of the *AtHSD1* was involved in regulating plant growth and development [41]. However, the above reports pertained to the expression of *AtHSD1* via nucleus. In contrast, it is interesting to see the modulation of plant growth regulation by expression of the *3β-HSD*, *P5βR1* and *P5βR2* genes in plastids.

### 3.2. Enhanced Biosynthesis of Glutamate, Glutamine, Proline and Sucrose in Transplastomic Plants under Salt Stress

The metabolites content was determined by NMR in four-week salt (300 mM NaCl) stressed WT and transplastomic plants. Among the metabolites, levels of glutamate, glutamine, proline and sucrose were enhanced in transplastomic plants compared to WT under salt stress. Glutamine synthetase (GS), a fundamental enzyme in N assimilation and remobilization, constructs the GS-GOGAT cycle with glutamate synthase (GOGAT) to convert inorganic ammonium into glutamine. The GS exists as isoforms: the cytosolic GS1 and the plastidic GS2. Cytosolic GS1 is responsible for primary ammonium assimilation in the roots or re-assimilation of ammonium produced in the leaves during protein turnover. GS2 is mainly accountable for assimilation of ammonium produced from photorespiration in chloroplasts [42].

In response to salinity and nitrogen (N) nutrition, various N metabolisms have been reported in several plant species [43,44]. For example, the nitrogen supply conferred salt tolerance to durum wheat cultivars (*Triticum turgidum* subsp. *durum*) [44]. Previous investigations also established that nitrogen metabolism and ion balance is altered due to salt stress in rice (*Oryza sativa* L.) [45]. Salt stress not only obstructs NO^3−^ uptake but also reduces N assimilation by hindering the production and actions of N assimilation enzymes including glutamine synthetase (GS), and glutamate synthase (GOGAT) [46]. It is also evident that an ample amount of nitrogen supply amends nutritional shortcoming in salt-stressed plants [47]. There is also growing evidence that the supply of N fertilizers could ameliorate the salt stress in plants [48,49,50,51,52,53,54]. Both glutamine and glutamate are good indicators of efficient nitrogen utilization [55]. In our study, the marked increase in amino acids, specially glutamine and glutamate in transplastomic lines as compared to WT, indicates possible enhanced nitrogen assimilation and thus enhanced salt tolerant phenotypes (Figure 5 and Figure 6A). Therefore, we speculate that higher level of glutamate and glutamine enhanced efficiency of N assimilation in transplastomic seedlings, which improved growth under salt stress.

**Figure 7:**
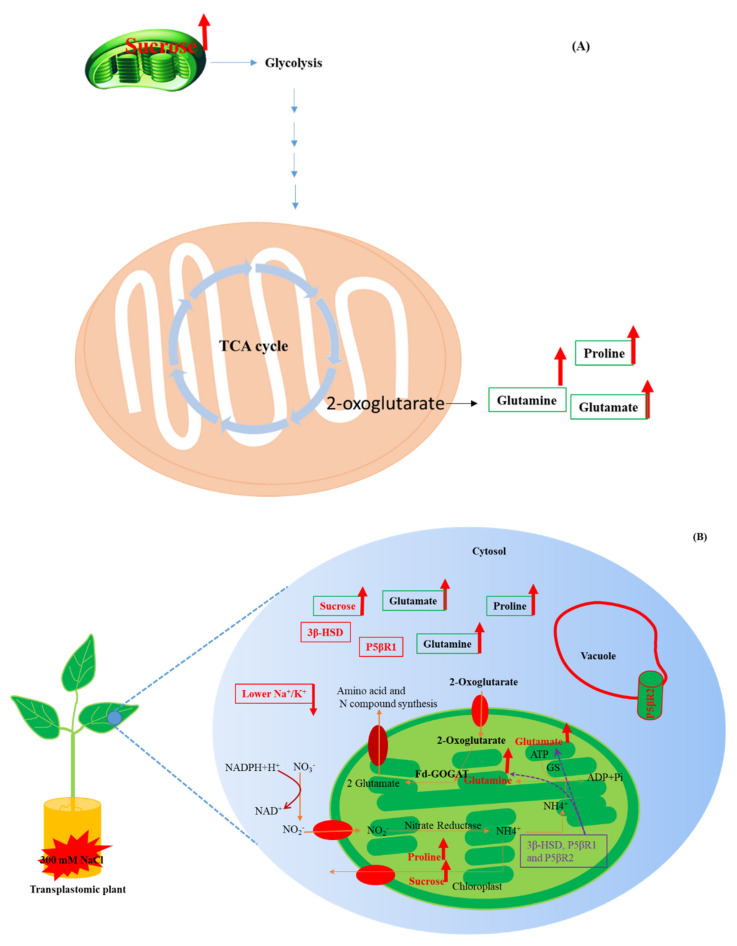
Improvement of salt tolerance in salt-stressed transplastomic plants of the *3β-HSD*, *P5βR1* and *P5βR2* due to enhanced synthesis of carbon-nitrogen skeleton metabolites and ionic balance. (**A**) Depicts the synthesis of carbon-nitrogen skeleton related metabolites due to induction of 300 mM NaCl in transplastomic plants. The genes *3β-HSD*, *P5βR1* and *P5βR2* seems to play an efficient role in synthesis of glutamate, glutamine, proline and sucrose. (**B**) Subcellular localization of the *3β-HSD*, *P5βR1* and *P5βR2* genes in cell and the accumulation of metabolites (sucrose, glutamate, glutamine and proline) or ionic balance (Na^+^/K^+^) in the cell as a mechanism for salt tolerance. Bold red arrows upward show the metabolites which were enhanced and the bold red arrow downward represent decreased level of ion content under salt stress.

We report that the plants overexpressing *SDR* genes display increased growth in terms of root length and FW. Our results indicate that the higher biomass production is supported by higher sucrose levels, as well as by possible changes in carbon and nitrogen metabolism. Likewise, our results related to metabolite analyses in leaves show that the overexpression of the *SDR* genes would trigger manifold changes in carbon-skeleton production and nitrogen assimilation pathways (Figure 7). Higher levels of proline and sucrose in transplastomic plants can improve osmotic adjustment under salinity efficiently than in WT plants. The increased growth observed could be associated to higher chlorophyll content, which lead to higher sucrose levels, and possibly enhanced nitrogen assimilation.

The amino acids glutamine and glutamate are also involved to synthesize other organo-nitrogen compounds such as nucleotides, chlorophyll, and also other amino acids like proline (Figure 7A) [56,57]. Renau-Morata et al. [58] demonstrated that over-expression of the *AtCDF3* (*Arabidopsis thaliana* Cycling DOF Factor3) supported the synthesis of sucrose eventually available for plant growth and development which ultimately increased level of glutamate and glutamine amino acids associated to nitrogen (N) assimilation. There are evidences that salt stresses induce the production and accumulation of glutamate and glutamine and elevates the activity of glutamate synthase and glutamine synthase [59,60,61]. Toxic NaCl levels can have impact on plant metabolism via interrupting nitrogen assimilation pathway, therefore decreasing the nitrogen level in the plant [60,62,63]. It is also possible that due to competition or antagonistic effect among N and NaCl, transplastomic plants could uptake more N or efficiently assimilate to ameliorate the toxic effect of NaCl, while WT plants could not do so. In this study, higher levels of sucrose and other amino acids indicate increased nitrogen assimilation as well as maintenance of chlorophyll content in transplastomic plants under salt stress. Various metabolites such as sugars and proline accumulate in the plant cells due to abiotic stresses such as salt (reviewed by [64,65]. These osmolytes work in a variety of ways, such as the protection of cellular structures, detoxification of the enzymes and scavenging of ROS alone or in combination with other defense-related enzyme systems [66,67]. These compounds provide integrity to the membranes [67] and keep the photosynthetic system functioning, as evident in the present study from the enhanced level of chlorophyll content in transplastomic plants compared to WT. Thus, a higher level of osmolytes such as proline and sucrose in transplastomic plants might confer salt tolerance and also protect cellular components, enhanced energy metabolism, detoxification of enzymes and reducing toxic reaction oxygen species [67,68,69].

### 3.3. 3β-HSD, P5βR1 and P5βR2 Confer Salt Tolerance

In the present study, it was observed that the transplastomic expression of the *3β-HSD*, *P5βR1* and *P5βR2* confer NaCl tolerance in tobacco plants, which is strengthened by two major proofs: (1) maintaining higher growth, biomass and chlorophyll in the transplastomic lines under NaCl treatment in comparison to WT (Figure 4 and Figure 5, Supplementary Figures S2A–C, S3 and S4); (2) higher biosynthesis of sucrose, glutamate, glutamine and proline in transplastomic lines under salt stress than that in WT (Figure 6A). Compared to WT, the higher growth and biomass due to NaCl treatment in transplastomic lines and the synthesis of higher metabolites seems firmly associated, which apparently attributable to higher levels of the *3β-HSD*, *P5βR1* and *P5βR2* transcripts in transplastomic lines compared to WT. These results suggest that the *3β-HSD*, *P5βR1* and *P5βR2* could be among the candidate genes for development of NaCl tolerant crops. The inducible biosynthesis of metabolites (sucrose, glutamate, glutamine and proline) due to NaCl stress could play a more vital role in conferring NaCl tolerance in transplastomic plants than in WT. The production of these metabolites has also documented previously due to NaCl inducing effect [53,54,55,56]. Overexpression of the *AtHSD1* enhanced growth under normal growth condition and tolerance to NaCl was reported due to ABA metabolism and production of BR-like effects [36]. In our results, plastidial expression of the *3β-HSD*, *P5βR1* and *P5βR2* (orthologous of the *AtHSD1*) could induce synthesis of nitrogen metabolism related compounds such as proline, glutamate and glutamine and such evidence not yet reported for the *SDR* family genes.

Plants have evolved a variety of mechanisms to deal with salt stress, and compartmentalization of Na^+^ into the vacuole is one of the most important mechanisms for maintaining a low Na^+^ content in the cytoplasm [70]. Vacuolar Na^+^ compartmentalization not only keeps Na^+^ away from the cytosolic components but also averts its deleterious effect [71]. Therefore, we speculate that transplastomic lines encompassing the highest amount of Na^+^ in leaf tissues (Supplementary Figure S5) could compartmentalize into vacuole or unknown organelles efficiently, thus protecting photosynthetic machinery and cytosolic components from toxic level of Na^+^. The Na^+^/K^+^ ratio (Figure 6B) was detected in the transplastomic tobacco study, which showed that the *3β-HSD*, *P5βR1* and *P5βR2* overexpressing plants had higher K^+^ content (Supplementary Figure S6) and a better-balanced Na^+^/K^+^ ratio to reduce salt-stress symptoms [72]. Further, there is a growing evidence that the *SDR* gene family has pivotal role in salinity stress tolerance in microbes [34] and plants [36,73] but not a single evidence via plastidial expression. Therefore, here we report the transplastomic expression of the *SDR* gene family member in tobacco. The evidence demonstrates that *SDR* gene family members localized to cytoplasm (3β-HSD and P5βR1) or (P5βR2) vacuolar membrane expressed via transplastomic expression confer salt tolerance in tobacco.

Thus, the *3β-HSD*, *P5βR1* and *P5βR2* genes seem to play a critical role in normal plant growth and development and under toxic levels of salinity. The *3β-HSD*, *P5βR1* and *P5βR2* genes could ameliorate deleterious effect of sodium salinity by upregulating the osmo-protectants as well as nitrogen metabolism compounds, and also maintaining the balanced ratio of Na^+^/K^+^ in leaf tissues. The transplastomic expression of the *3β-HSD*, *P5βR1* and *P5βR2* genes could target multiple pathways such as carbon (sucrose) nitrogen skeleton (glutamate, glutamine and proline), ion balance (Na^+^/K^+^) (Figure 6 and Figure 7) rather than single pathway for salinity tolerance induction.

### 3.4. Protective Role of Proline, Sucrose, Glutamate and Glutamine under Salt Stress

It was interesting to observe an increase in the number of metabolites produced in transplastomic plants in comparison to untransformed wild-type tobacco plants. This increase in the levels of proline, sucrose, glutamate and glutamine can be directly correlated with the enhanced salt tolerance in tobacco plastome transformed plants. Proline is an amino acid, which is a source of nitrogen compound protects plant cells under salt stress. It plays role in variety of ways such as protecting enzymes, cellular structures, reducing oxidative stress by acting as free radical scavenger and promoting cellular water retention [74,75]. Sucrose/sugars not only provide energy but also contribute to the regulation of ROS signaling as well as osmotic adjustments during abiotic stresses [76]. Further, soluble sugars are also involved in protection of mitochondrial respiration and photosynthesis [77]. Glutamate and glutamine accumulate under salt stress and act as salt tolerant mechanisms in plants [78]. Amino acid (proline, glutamate and glutamine) accumulation may be considered as a detoxification mechanism of the ammonium produced in plants subjected to stress [79,80]. Proline synthesis occurs in mitochondria but accumulates in cytosol under salt stress in plant. Sucrose synthesis takes place in mesophyll/chloroplast and transported to cytosol. Glutamate and glutamine are also synthesized in chloroplast and accumulates in cytosol.

The transplastomic expression of three genes of the *SDR* gene family conferred enhanced salt tolerance in tobacco plants. Thus, in this report, and for the first time, we are reporting on the expression of the *SDR* gene family members in plastid genome. The present study forms a basis for the development of salt-tolerant plants via plastid genetic engineering.

## 4. Materials and Methods

### 4.1. Vector Construction

The *3β-HSD*, *P5βR1* and *P5βR2* genes (NCBI accession no. KM406483.1, KJ766303, GU062787) were selected for expression in plastid genomes of *Nicotiana tabacum*. The 3*β-HSD*, *P5βR1* and *P5βR2* genes were amplified from *Digitalis ferruginea* subsp. *ferruginea* [37]. Fresh leaf samples of *D. ferruginea* subsp. *ferruginea* (100 mg) were ground to a fine powder using liquid nitrogen with mortar and pestle. Total RNA isolation was carried out with GeneJET Plant RNA Purification Kit (ThermoFischer Scientific, Waltham, MA, USA). Samples were treated with RNase free DNase I to remove genomic DNA contamination. Single strand cDNA was synthesized by reverse transcription-polymerase chain reaction (RT-PCR) using SuperScriptTM III RT-PCR kit according to the instruction recommended by manufacturer (ThermoFischer Scientific, Waltham, MA, USA). Purified total RNA up to 5 µg was used to synthesize cDNA. Phusion^®^ High-Fidelity DNA polymerase (NEB, Ipswich, MA, USA) was used for amplification of the *3β-HSD*, *P5βR1* and *P5βR2* genes by using gene specific primers as follows: 3β-HSD forward primer: 5′-GGGGACAAGTTTGTACAAAAAAGCAGGCTTAATGTCGTCAAAGCCAAGGTTGG-3′, 3β-HSD reverse primer: 5′-GGGGACCACTTTGTACAAGAAAGCTGGGTTCTAACGCACGACGGTGAAGC-3′, P5βR1 forward primer: 5′-GGGGACAAGTTTGTACAAAAAAGCAGGCTTAATGAGCTGGTGGTGGGC-3′, P5βR1 reverse primer: 5′-GGGGACCACTTTGTACAAGAAAGCTGGGTTAGGAACAATCTTGTAAGCTTTTGCCT-3′, P5βR2 forward primer 5′-GGGGACAAGTTTGTACAAAAAAGCAGGCTTAATGTATACCGACACAACGACTTGG-3′ and P5βR2 reverse primer: 5′-GGGGACCACTTTGTACAAGAAGCTGGGTTAGGGACAAATCTATAAGTTCTCACTTTGT-3′. Primers used in the present study are also summarized in Supplementary Table S1. The amplified products were confirmed on 1% agarose gel stained with EtBr and further confirmed by sequencing. For construction of final plastid transformation vector, Gateway^®^ cloning was used. The genes *3β-HSD*, *P5βR1* and *P5βR2* were cloned into pDONR221 by BP recombination reaction which resulted in entry vectors pENTR-3β-HSD, pENTR-P5βR1 and pENTR-P5βR2. An LR recombination reaction was carried out between pENTR-3β-HSD, pENTR-P5βR1 and pENTR-P5βR2 and pDEST-PN-T in separate reaction for each entry vector and final plastid expression vectors pEXP-PN-T-3β-HSD, pEXP-PN-T- P5βR1 and pEXP-PN-T-P5βR2 were obtained. A plastid specific Gateway^®^ compatible destination vector, pDEST-PN-T [81], was used for the LR reaction. It contained the cassette of *aadA* gene under the control of *psbA* promoter (P*psbA*), the 5′UTR of tobacco *psbA* gene (5′psbA) and the 3′UTR from large subunit of ribulose-bisphosphate carboxylase gene (*rbc*L) from *Chlamydomonas reinhardtii*. The expression of transgene was under the control of constitutive P*rrn*PEP+NEP promoter, which consisted of the nuclear encoded polymerase (P*rrn*-62NEP) promoter [82] fused downstream to the plastid-encoded polymerase (PEP) promoter P*rrn*16 [83]. Figure 1 shows the vector construction steps. The Gateway^®^ cloning kit was purchased from (ThermoFischer Scientific, Waltham, MA, USA) and all cloning reactions were carried out by following the instructions of manufacturer.

### 4.2. Plastid Transformation of Tobacco and Regeneration of Transformed Plants

The plastid transformation was carried out by following the procedure as described previously [84]. Briefly, seeds of *N*. *tabacum* (Nt) cv. Petit Havana were grown in vitro at 26 °C on agar solidified MS [85] medium containing 3% sucrose. The expression constructs, pEXP-PN-T-3β-HSD, pEXP-PN-T-P5βR1 and pEXP-PN-T-P5βR2, were coated onto gold particles of 0.6 µm and bombarded on 2 weeks old tobacco leaves by bombarding DNA coated gold particles using particle gun (PDS1000He; Bio-Rad, Hercules, CA, USA). After bombardment, leaves were sliced into small pieces of 5 mm and placed on RMOP media containing 500 mg/L spectinomycin [30] for the selection and regeneration. After 2–3 weeks, green calli emerged which were further developed into shoots. The shoots were cut into small pieces and then placed again on RMOP medium containing the antibiotic. The procedure was repeated 3–4 times to get homoplasmy of the transformed shoots. The tissues of the shoots were harvested to isolate DNA and RNA for the confirmation of integration of transgene into tobacco plastid genome and to check expression level of the transgene, respectively. After the confirmation of transgene, the transgenic seedlings were transferred to rooting medium. After one month when fully developed roots were established, the plantlets were transferred to soil in green house for further growth and seed production.

### 4.3. Confirmation of Transformation and Transgene Expression

Total DNA from WT (wild type) as well as transplastomic plants was isolated using the hexadecyltrimethyl ammonium bromide (CTAB) method [86]. This DNA was used as template to perform PCR for confirming the presence of transgene. PCR was carried out to confirm the correct integration of the *3β-HSD* within the transplastomic plants by using sense primer 3βHSD_F (positioned within the *3β-HSD*; sequence 5′-ACGTCAGAGATGAAAAACAA-3′) and anti-sense primer oli252 (positioned within the chloroplast genome outside the right flank (*trn*R); sequence 5′-AGACAGCGACGGGTTCTCTG-3′). Correct insertion of the *P5βR1* gene within the transplastomic plants was confirmed by using sense primer P5βR1_F (5′-CCCATGATCCACCCTACA-3′) located within the *P5βR1* and anti-sense primer oli252 (5′- AGACAGCGACGGGTTCTCTG-3′) located within the chloroplast genome outside the right flank (*trn*R). Correct integration of the *P5βR2* gene within the transplastomic plants was confirmed by using sense primer P5βR2_F (5′-TTAGACAACCTAATTTCTATTACAATCTAGAAG-3′) positioned within the *P5βR2* gene and anti-sense primer oli252 (5′-AGACAGCGACGGGTTCTCTG-3′) located within the chloroplast genome outside of the right flank (*trn*R). Similarly, Correct insertion of the *aadA* gene within the transplastomic plants containing *3β-HSD*, *P5βR1* and *P5βR2*, was confirmed by using sense primer oli253 (5′-GATCCGAGCCATAGAATTTC-3′) located in the chloroplast genome outside of the left flank (*trn*N) and anti-sense primer oli059 (5′-TGCTGGCCGTACATTTGTACG-3′) located within the *aadA* gene. The positions of primers and expected fragment sizes are shown in (Figure 1).

### 4.4. Confirmation of Transgene Expression by Real Time Qrt-PCR

Transgene expression was determined by Real-Time Quantitative Reverse Transcription PCR (RT-qRT-PCR). Similar procedure was performed for the isolation of RNA and cDNA synthesis from transplastomic and WT plants as described previously [87]. For RT-qRT-PCR, gene-specific primer sets for the *3βHSD*; 3βHSD-F 5′-GCTTACACGGCTTCCAAACA-3′, 3βHSD-R 5′-CCCTTCAAGTTAGCCCTGGA-3′, *P5βR1*; P5βR1-F 5′-TGCAAACACGAGGGAAAGGT-3′, P5βR1-R 5′-TCTACTCCAAACTGCTCCGC-3′, *P5βR2*; P5βR2-F 5′-GGACAGAAACGTCGTGGAAT-3′, P5βR2-R 5′-CGTCCCATACCGAGTCCTTA-3′ were used. *Actin9* was used as reference gene as described previously [88]. Conditions for real time PCR were: 95 °C for 30 s; 40 cycles at 95 °C for 10 s, 60 °C for 30 sec and 72 °C for 15 s. to amplify the genes, using a SsoAdvanced Universal SYBR Green Supermix (Bio-Rad, Hercules, CA, USA) by CFX Connect™ Real-Time PCR Detection System (Bio-Rad, Hercules, CA, USA). The gene expression was calculated as explained previously [89].

### 4.5. End-to-End PCR

End-to-end PCR for detecting whether all plastomes are in transformed state. The method has been used previously for investigating that all plastid genomes are in transformed state and no wild-type plastid genome is left [90,91]. For this purpose, a pair of primers was used which gave positive results for both wild-type and transgenic lines with different amplicon sizes. Sense primer, oli253, was located within plastome outside *trn*N and anti-sense primer oli252 was located within the *trn*R. The sequences of these primers were: oli253 (5′-GATCCGAGCCATAGAATTTC-3′) and oli252 (5′-AGACAGCGACGGGTTCTCTG-3′). The standard PCR reaction conditions were used and Tm of the primers was 52 °C. The positions of these primers and expected fragment sizes are shown in (Figure 1).

### 4.6. Western Blot

Immunoblot analysis was carried out following the procedure with minor modifications as described [87]. Protein was extracted from the leaves of transplastomic and wild-type plants. To extract the total soluble protein from transplastomic and wild-type plants, approximately 100 mg of leaves were ground thoroughly in liquid nitrogen and then homogenized in a protein extraction buffer containing: 0.5 M sorbitol, 10 mM ethylene glycol tetra acetic acid (EGTA), 10 mM sodium orthovanadate (Na_3_VO_4_), 10 mM sodium fluoride (NaF), 5% (*v*/*v*) polyvinylpyrrolidone, 25 mM 4-(2-hydroxyethyl)-1-piperazineethanesulfonic acid-BTP (HEPES-BTP) (pH 7.6) and five additional components [0.5% (*w*/*v*) bovine serum albumin (protease free, A-3294 obtained from Sigma, St. Louis, MO, USA), 1 mM dithiothreitol, 0.5 mM phenylmethyl sulfonyl fluoride, 5 µg/mL leupeptin, and 0.5 µg/mL pepstatin A] were added just before use. The homogenized samples were centrifuged at 14,000 g for 10 min at 4 °C and the supernatants were collected as soluble fraction. Soluble proteins were quantified by the Bradford method [92]. Fifteen µg of soluble fractions were loaded in 12% polyacrylamide SDS-PAGE. Primary antibodies of anti-3βHSD, anti-P5βR1 and anti-P5βR2 were raised against the peptide sequences: from 238 to 255 SDESAYVSGQNLAVDGGF, from 372 to 389 KNAFISWIDKAKAYKIVP and from 375 to 394 DSTKSFISSVNKVRTYRFVP, respectively (ThermoFischer Scientific; http://www.pierce-antibodies.com/). The blotted membranes were probed with the anti-polyclonal antibodies. The secondary antibody treatment was done with the WesternSure^®^ HRP Goat anti-Rabbit IgG (LI-COR Biosciences, Lincoln, NE, USA) for 1 h, and Clarity™ and Clarity Max™ Western ECL Substrates (BioRad, Hercules, CA, USA) was used for chemiluminescence for 5 min, which was then observed under detection system (C-DiGit Chemiluminescent Western Blot Scanner, LI-COR Biosciences, Lincoln, NE, USA).

### 4.7. Cloning of the 3βhsd, P5βr1 and P5βr2 and Vector Construction for Subcellular Localization

In order to determine the function of the genes, the construction of expression vectors for subcellular localization was carried out, using the expression plasmid pGWB5 [93] following the procedure as described previously with few modifications [87]. RNA isolation and cDNA synthesis were performed as described earlier in the section of Vector Construction. Phusion^®^ High-Fidelity DNA polymerase (NEB, USA) was used for the amplification of the *3βHSD*, *P5βR1* and *P5βR2* genes using gene specific primers as given below: 3βHSD-F 5′-GGGGACAAGTTTGTACAAAAAAGCAGGCTTAatgtcgtcaaagccaaggttgg-3′, 3βHSD-R 5′-GGGGACCACTTTGTACAAGAAAGCTGGGTTacgcacgacggtgaagc-3′, P5βR1-F 5′-GGGGACAAGTTTGTACAAAAAAGCAGGCTTAatgagctggtggtgggc-3′, P5βR2-R 5′-GGGGACCACTTTGTACAAGAAAGCTGGGTTaggaacaatcttgtaagcttttgcct-3′, P5βR2-F5′-GGGGACAAGTTTGTACAAAAAAGCAGGCTTaatgtataccgacacaacgacttgg-3′, P5βR2-R5-′GGGGACCACTTTGTACAAGAAAGCTGGGTTagggacaaatctataagttctcactttgttaac-3′. The products were used for the Gateway BP and LR reactions following the instructions of the manufacturer (ThermoFischer Scientific, Waltham, MA, USA). Three plasmid constructs were developed: 3β-HSD::GFP, P5βR1::GFP and P5βR2::GFP by Gateway cloning. These were transformed into *A*. *tumefaciens* strain C58C1. For subcellular localization, *Agrobacterium*-mediated transformation into onion epidermal cells was performed according to the protocol described by [94]. Transient expression of the genes in onion epidermal cells was observed under the confocal laser microscope system C2si (Nikon, Minato, Tokyo, Japan).

### 4.8. Salt Tolerance Assessment and NMR-Based Metabolites Determination

To monitor the effect of salt stress on seedlings, WT and transplastomic seedlings were grown on MS medium containing no (control; CK) or different concentrations of NaCl (50 mM, 200 mM, 300 mM) in sealed petri plates. The WT and transplastomic seeds were germinated on MS [85] agar solidified media without NaCl for 7 days. The seven-day-old seedlings were transferred to square Petri plates containing MS [85] agar media and various concentration (0 mM, 50 mM, 200 mM and 300 mM) of NaCl. The plates were placed in growth room in vertical position. After one month, the primary and lateral root length and biomass was measured using scale or weighing balance. After one month the primary and lateral root length and biomass was measured. Chlorophyll content was also measured in the treatment of 300 mM NaCl. Further, the metabolome analysis by NMR [95] was performed using the seedlings from CK and 300 mM NaCl treatments. Na^+^ and K^+^ concentration in leaf of 300 mM NaCl treated seedlings for one month was determined by following the procedure as previously described [96]. 

The osmotic stress was imposed by following the procedure [97]. Briefly, one-week-old seedlings of WT and transplastomic lines were used for the osmotic stress assay by exposing to 400 mM mannitol. Roots of the seven-day-old seedlings were excised and the shoots were cultured on solid MS media (containing 0.5% sucrose) supplemented with 400 mM concentration of mannitol. After 25 days, the root length, number of lateral roots and fresh weight of the shoots were measured. For salt stress three repeats and the number of plants (WT *n* = 3, transplastomic lines *n* = 8 for each repeat of each line) were employed. For osmotic stress, the number of seedlings (*n* = 5) for each line of each repeat were used. The experiment was performed at least twice. For NMR analysis leaves of 3 plants of each treatment or lines were randomly collected and ground in liquid nitrogen to a fine powder. After extraction of the content with suitable NMR solvent and buffer, triplicate samples were prepared from the extract and stored at −20 °C prior to analysis. Three plant extracts were analyzed separately in NMR under certain conditions and there were very slight variations (insignificant) in the proton NMR results so the findings are given as one result without using an error bar. A one-way ANOVA test was performed following LSD test at *p* < 0.05.

## Data Availability

Data is contained in Supplementary Materials.

## Supplementary Materials

The following are available online at https://www.mdpi.com/article/10.3390/ijms222111736/s1.

## References

[1] Bertol J., Rigotto C., de Pádua R.M., Kreis W., Barardi C.R.M., Braga F., Simões C.M.O. (2011). Antiherpes activity of glucoevatromonoside, a cardenolide isolated from a Brazilian cultivar of Digitalis lanata. Antivir. Res..

[2] Kreis W., Haug B., Yucesan B. (2014). Somaclonal variation of cardenolide content in Heywood’s foxglove, a source for the antiviral cardenolide glucoevatromonoside, regenerated from permanent shoot culture and callus. Vitr. Cell. Dev. Biol. Anim..

[3] Pérez-Bermúdez P., Moya García A.A., Tuñón I., Gavidia I. (2010). *Digitalis purpurea* P5βR2, encoding steroid 5β-reductase, is a novel defense-related gene involved in cardenolide biosynthesis. N. Phytol..

[4] Kreis W. (2017). The Foxgloves (Digitalis) Revisited. Planta Med..

[5] Ernst M., de Padua R.M., Herl V., Muller-Uri F., Kreis W. (2010). Expression of 3beta-HSD and P5betaR, genes respectively coding for Delta5-3beta-hydroxysteroid dehydrogenase and progesterone 5beta-reductase, in leaves and cell cultures of Digitalis lanata EHRH. Planta Med..

[6] Herl V., Fischer G., Müller-Uri F., Kreis W. (2006). Molecular cloning and heterologous expression of progesterone 5β-reductase from Digitalis lanata Ehrh. Phytochemistry.

[7] Herl V., Frankenstein J., Meitinger N., Muller-Uri F., Kreis W. (2007). Delta 5-3beta-hydroxysteroid dehydrogenase (3 beta HSD) from Digitalis lanata. Heterologous expression and characterisation of the recombinant enzyme. Planta Med..

[8] Cacho M., Morán M., Tárrago J.F., Corchete P. (1995). Calcium restriction induces cardenolide accumulation in cell suspension cultures of *Digitalis thapsi* L. Plant Cell Rep..

[9] Cingoz G.S., Gurel E. (2016). Effects of salicylic acid on thermotolerance and cardenolide accumulation under high temperature stress in Digitalis trojana Ivanina. Plant Physiol. Biochem..

[10] Paranhos A., Fernández-Tárrago J., Corchete P. (1999). Relationship between active oxygen species and cardenolide production in cell cultures of Digitalis thapsi: Effect of calcium restriction. N. Phytol..

[11] Finsterbusch A., Lindemann P., Grimm R., Eckerskorn C., Luckner M. (1999). Delta(5)-3beta-hydroxysteroid dehydrogenase from Digitalis lanata Ehrh.—A multifunctional enzyme in steroid metabolism?. Planta.

[12] Saito K., Yamazaki M., Shimomura K., Yoshimatsu K., Murakoshi I. (1990). Genetic transformation of foxglove (*Digitalis purpurea*) by chimeric foreign genes and production of cardioactive glycosides. Plant Cell Rep..

[13] Saito K., Yamazaki M., Kaneko H., Murakoshi I., Fukuda Y., Van Montagu M. (1991). Tissue-specific and stress-enhancing expression of the TR promoter for mannopine synthase in transgenic medicinal plants. Planta.

[14] Lehmann U., Moldenhauer D., Thomar S., Diettrich B., Luckner M. (1995). Regeneration of plants from Digitalis lanata cells transformed with Agrobacterium tumefaciens carrying bacterial genes encoding neomycin phosphotransferase II and β-glucuronidase. J. Plant Physiol..

[15] Li Y., Gao Z., Piao C., Lu K., Wang Z., Cui M.-L. (2014). A stable and efficient Agrobacterium tumefaciens-mediated genetic transformation of the medicinal plant *Digitalis purpurea* L. Appl. Biochem. Biotechnol..

[16] Pérez-Alonso N., Chong-Pérez B., Capote A., Pérez A., Izquierdo Y., Angenon G., Jiménez E. (2014). Agrobacterium tumefaciens-mediated genetic transformation of *Digitalis purpurea* L. Plant Biotechnol. Rep..

[17] Pradel H., Dumke-Lehmann U., Diettrich B., Luckner M. (1997). Hairy root cultures of Digitalis lanata. Secondary metabolism and plant regeneration. J. Plant Physiol..

[18] Sales E., Segura J., Arrillaga I. (2003). Agrobacterium tumefaciens-mediated genetic transformation of the cardenolide-producing plant *Digitalis minor* L.. Planta Med..

[19] Sales E., Munoz-Bertomeu J., Arrillaga I., Segura J. (2007). Enhancement of cardenolide and phytosterol levels by expression of an N-terminally truncated 3-hydroxy-3-methylglutaryl CoA reductase in Transgenic digitalis minor. Planta Med..

[20] Shi H.P., Lindemann P. (2006). Expression of recombinant Digitalis lanata EHRH. cardenolide 16′-O-glucohydrolase in Cucumis sativus L. hairy roots. Plant Cell Rep..

[21] Hagimori M., Matsumoto T., Obi Y. (1982). Studies on the Production of Digitalis Cardenolides by Plant Tissue Culture: II. Effect of light and plant growth substances on digitoxin formation by undifferentiated cells and shoot-forming cultures of digitalis purpurea l. Grown in liquid media. Plant Physiol..

[22] Waheed M.T., Ismail H., Gottschamel J., Mirza B., Lössl A.G. (2015). Plastids: The green frontiers for vaccine production. Front.plant sci..

[23] Daniell H. (2002). Molecular strategies for gene containment in transgenic crops. Nat. Biotechnol..

[24] Hagemann R., Daniell H., Chase C. (2004). The Sexual Inheritance of Plant Organelles. Molecular Biology Biotechnol. Plant Organelles: Chloroplasts and Mitochondria.

[25] Munns R. (2005). Genes and salt tolerance: Bringing them together. N. Phytol..

[26] Zhu J.-K. (2001). Plant salt tolerance. Trends Plant Sci..

[27] Munns R., Tester M. (2008). Mechanisms of salinity tolerance. Annu. Rev. Plant Biol..

[28] Yamaguchi T., Blumwald E. (2005). Developing salt-tolerant crop plants: Challenges and opportunities. Trends Plant Sci..

[29] Gurel E., Karvar S., Yucesan B., Eker I., Sameeullah M. (2017). An overview of cardenolides in digitalis-more than a cardiotonic compound. Curr. Pharm. Des..

[30] Svab Z., Hajdukiewicz P., Maliga P. (1990). Stable transformation of plastids in higher plants. Proc. Natl. Acad. Sci. USA.

[31] Dangol S., Singh R., Chen Y., Jwa N.-S. (2017). Visualization of multicolored in vivo organelle markers for co-localization studies in Oryza sativa. Mol. Cells.

[32] Klein J., Horn E., Ernst M., Leykauf T., Leupold T., Dorfner M., Wolf L., Ignatova A., Kreis W., Munkert J. (2021). RNAi-mediated gene knockdown of progesterone 5β-reductases in Digitalis lanata reduces 5β-cardenolide content. Plant Cell Rep..

[33] Malcolm S.B., Zalucki M.P. (1996). Milkweed latex and cardenolide induction may resolve the lethal plant defence paradox. Èntomol. Exp. Appl..

[34] Pumirat P., Boonyuen U., Vanaporn M., Pinweha P., Tandhavanant S., Korbsrisate S., Chantratita N. (2014). The role of short-chain dehydrogenase/oxidoreductase, induced by salt stress, on host interaction of B. pseudomallei. BMC Microbiol..

[35] Yu S., Sun Q., Wu J., Zhao P., Sun Y., Guo Z. (2021). Genome-Wide Identification and Characterization of Short-Chain Dehydrogenase/Reductase (SDR) Gene Family in Medicago truncatula. Int. J. Mol. Sci..

[36] Zou C., Chen A., Xiao L., Muller H.M., Ache P., Haberer G., Zhang M., Jia W., Deng P., Huang R. (2017). A high-quality genome assembly of quinoa provides insights into the molecular basis of salt bladder-based salinity tolerance and the exceptional nutritional value. Cell Res..

[37] Eker İ., Yücesan B., Sameeullah M., Welβ W., Müller-Uri F., Gürel E., Kreis W. (2016). Phylogeny of Anatolian (Turkey) species in the Digitalis sect. Globiflorae (Plantaginaceae). Phytotaxa.

[38] Maathuis F.J.M. (2013). Sodium in plants: Perception, signalling, and regulation of sodium fluxes. J. Exp. Bot..

[39] Shabala S. (2003). Regulation of Potassium Transport in Leaves: From Molecular to Tissue Level. Ann. Bot..

[40] Cheng W.H., Endo A., Zhou L., Penney J., Chen H.C., Arroyo A., Leon P., Nambara E., Asami T., Seo M. (2002). A unique short-chain dehydrogenase/reductase in Arabidopsis glucose signaling and abscisic acid biosynthesis and functions. Plant Cell.

[41] Li F., Asami T., Wu X., Tsang E.W.T., Cutler A.J. (2007). A Putative Hydroxysteroid Dehydrogenase Involved in Regulating Plant Growth and Development. Plant Physiol..

[42] Thomsen H.C., Eriksson D., Møller I.S., Schjoerring J.K. (2014). Cytosolic glutamine synthetase: A target for improvement of crop nitrogen use efficiency?. Trends Plant Sci..

[43] Liu X., Xu C., Xu K., Cui J., Zhang Z.A., Ling F., An J., Wu Z. (2015). Effects of salt stress on photosynthetic characteristics and some physiological traits of rice varieties at different nitrogen levels. J. South China Agric. Univ..

[44] Nasraoui H.A., Bouthour D., Hfaidh R., Gouia H., Pageau K., Chaffei H.C. (2013). The role of nitrogen availability for the salt-tolerance of two different varieties of durum wheat. Bull. Environ. Contam. Toxicol..

[45] Wang H., Wu Z., Zhou Y., Han J., Shi D. (2012). Effects of salt stress on ion balance and nitrogen metabolism in rice. Plant Soil Environ..

[46] Hossain M., Uddin M., Ismail M.R., Ashrafuzzaman M. (2012). Responses of glutamine synthetase-glutamate synthase cycle enzymes in tomato leaves under salinity stress. Int. J. Agricul. Biol..

[47] Meng S., Su L., Li Y., Wang Y., Zhang C., Zhao Z. (2016). Nitrate and Ammonium Contribute to the Distinct Nitrogen Metabolism of Populus simonii during Moderate Salt Stress. PLoS ONE.

[48] Zhonghua T., Yanju L., Xiaorui G., Yuangang Z. (2011). The combined effects of salinity and nitrogen forms on Catharanthus roseus: The role of internal ammonium and free amino acids during salt stress. J. Plant Nutr. Soil Sci..

[49] Hessini K., Hamed K.B., Gandour M., Mejri M., Abdelly C., Cruz C. (2013). Ammonium nutrition in the halophyte Spartina alterniflora under salt stress: Evidence for a priming effect of ammonium?. Plant Soil.

[50] De Souza Miranda R., Gomes-Filho E., Prisco J.T., Alvarez-Pizarro J.C. (2016). Ammonium improves tolerance to salinity stress in Sorghum bicolor plants. Plant Growth Regul..

[51] Ding F., Wang R., Chen B. (2019). Effect of exogenous ammonium gluconate on growth, ion flux and antioxidant enzymes of maize (Zea Mays, L.) seedlings under NaCl stress. Plant Biol..

[52] Bezerra M.A.F., Pereira W.E., Bezerra F.T.C., Cavalcante L.F., da Silva Medeiros S.A. (2019). Nitrogen as a mitigator of salt stress in yellow passion fruit seedlingss. Semina Ciências Agrárias.

[53] Hessini K., Issaoui K., Ferchichi S., Saif T., Abdelly C., Siddique K.H.M., Cruz C. (2019). Interactive effects of salinity and nitrogen forms on plant growth, photosynthesis and osmotic adjustment in maize. Plant Physiol. Biochem..

[54] Ashraf M., Shahzad S.M., Imtiaz M., Rizwan M.S., Arif M.S., Kausar R. (2018). Nitrogen nutrition and adaptation of glycophytes to saline environment: A review. Arch. Agron. Soil Sci..

[55] Stitt M., Krapp A. (1999). The interaction between elevated carbon dioxide and nitrogen nutrition: The physiological and molecular background. Plant Cell Environ..

[56] Forde B.G., Lea P.J. (2007). Glutamate in plants: Metabolism, regulation, and signalling. J. Exp. Bot..

[57] Bernard S.M., Habash D.Z. (2009). The importance of cytosolic glutamine synthetase in nitrogen assimilation and recycling. N. Phytol..

[58] Renau-Morata B., Molina R.V., Carrillo L., Cebolla-Cornejo J., Sánchez-Perales M., Pollmann S., Domínguez-Figueroa J., Corrales A.R., Flexas J., Vicente-Carbajosa J. (2017). Ectopic Expression of CDF3 Genes in Tomato Enhances Biomass Production and Yield under Salinity Stress Conditions. Front. Plant Sci..

[59] Berteli F., Corrales E., Guerrero C., Ariza M.J., Pliego F., Valpuesta V. (1995). Salt stress increases ferredoxin-dependent glutamate synthase activity and protein level in the leaves of tomato. Physiol. Plant..

[60] Debouba M., Gouia H., Suzuki A., Ghorbel M.H. (2006). NaCl stress effects on enzymes involved in nitrogen assimilation pathway in tomato “Lycopersicon esculentum” seedlings. J. Plant Physiol..

[61] Saito T., Matsukura C., Sugiyama M., Watahiki A., Ohshima I., Iijima Y., Konishi C., Fujii T., Inai S., Fukuda N. (2008). Screening for γ-aminobutyric acid (GABA)-rich tomato varieties. J. Jpn. Soc. Hortic. Sci..

[62] Flores P., Botella M.Á., Cerdá A., Martínez V. (2004). Influence of nitrate level on nitrate assimilation in tomato (Lycopersicon esculentum) plants under saline stress. Can. J. Bot..

[63] Debouba M., Maâroufi-Dghimi H., Suzuki A., Ghorbel M.H., Gouia H. (2007). Changes in growth and activity of enzymes involved in nitrate reduction and ammonium assimilation in tomato seedlings in response to NaCl stress. Ann. Bot..

[64] Parvanova D., Ivanov S., Konstantinova T., Karanov E., Atanassov A., Tsvetkov T., Alexieva V., Djilianov D. (2004). Transgenic tobacco plants accumulating osmolytes show reduced oxidative damage under freezing stress. Plant Physiol. Biochem..

[65] Bartels D., Sunkar R. (2005). Drought and salt tolerance in plants. Crit. Rev. Plant Sci..

[66] Verma D.P.S., Zhang C., Singh B.K. (1999). Regulation of proline and arginine biosynthesis in plants. Plant Amino Acids: Biochemistry and Biotechnology.

[67] Xiong L., Schumaker K.S., Zhu J.-K. (2002). Cell signaling during cold, drought, and salt stress. Plant Cell.

[68] Singh M., Kumar J., Singh S., Singh V.P., Prasad S.M. (2015). Roles of osmoprotectants in improving salinity and drought tolerance in plants: A review. Rev. Environ. Sci. Biotechnol..

[69] Hayat S., Hayat Q., Alyemeni M.N., Wani A.S., Pichtel J., Ahmad A. (2012). Role of proline under changing environments: A review. Plant Signal. Behav..

[70] Tester M., Davenport R. (2003). Na+ tolerance and Na+ transport in higher plants. Ann. Bot..

[71] Blumwald E. (2000). Sodium transport and salt tolerance in plants. Curr. Opin. Cell Biol..

[72] Jiang Z., Song G., Shan X., Wei Z., Liu Y., Jiang C., Jiang Y., Jin F., Li Y. (2018). Association Analysis and Identification of ZmHKT1;5 Variation With Salt-Stress Tolerance. Front. Plant Sci..

[73] Meng F., Luo Q., Wang Q., Zhang X., Qi Z., Xu F., Lei X., Cao Y., Chow W.S., Sun G. (2016). Physiological and proteomic responses to salt stress in chloroplasts of diploid and tetraploid black locust (Robinia pseudoacacia L.). Sci. Rep..

[74] Hong Z., Lakkineni K., Zhang Z., Verma D.P.S. (2000). Removal of Feedback Inhibition of Δ1-Pyrroline-5-Carboxylate Synthetase Results in Increased Proline Accumulation and Protection of Plants from Osmotic Stress1. Plant Physiol..

[75] Liu J., Zhu J.K. (1997). Proline Accumulation and Salt-Stress-Induced Gene Expression in a Salt-Hypersensitive Mutant of Arabidopsis. Plant Physiol..

[76] Vinocur B., Altman A. (2005). Recent advances in engineering plant tolerance to abiotic stress: Achievements and limitations. Curr. Opin. Biotechnol..

[77] Couée I., Sulmon C., Gouesbet G., El Amrani A. (2006). Involvement of soluble sugars in reactive oxygen species balance and responses to oxidative stress in plants. J. Exp. Bot..

[78] Ali Q., Habib-ur-Rehman Athar M.Z., Haider S.S., Aslam N., Shehzad F., Naseem J., Ashraf R., Ali A., Hussain S.M. (2019). Role of Amino Acids in Improving Abiotic Stress Tolerance to Plants. Plant Tolerance to Environmental Stress.

[79] Slocum R., Weinstein L. (1990). Osmotic stress-induced putrescine accumulation as a mechanism of ammonia detoxification in oat leaves. Plant Physiol. Supp..

[80] El-Bassiouny H.M., Bekheta M. (2005). Effect of salt stress on relative water content, lipid peroxidation, polyamines, amino acids and ethylene of two wheat cultivars. Int. J. Agricul. Biol..

[81] Gottschamel J., Waheed M.T., Clarke J.L., Lossl A.G. (2013). A novel chloroplast transformation vector compatible with the Gateway((R)) recombination cloning technology. Transgenic Res..

[82] Hajdukiewicz P.T., Allison L.A., Maliga P. (1997). The two RNA polymerases encoded by the nuclear and the plastid compartments transcribe distinct groups of genes in tobacco plastids. EMBO J..

[83] Svab Z., Maliga P. (1993). High-frequency plastid transformation in tobacco by selection for a chimeric aadA gene. Proc. Natl. Acad. Sci. USA.

[84] Waheed M.T., Thones N., Muller M., Hassan S.W., Gottschamel J., Lossl E., Kaul H.P., Lossl A.G. (2011). Plastid expression of a double-pentameric vaccine candidate containing human papillomavirus-16 L1 antigen fused with LTB as adjuvant: Transplastomic plants show pleiotropic phenotypes. Plant Biotechnol. J..

[85] Murashige T., Skoog F. (1962). A Revised Medium for Rapid Growth and Bio Assays with Tobacco Tissue Cultures. Physiol. Plant..

[86] Doyle J.J., Doyle J.L. (1987). A rapid DNA isolation procedure for small quantities of fresh leaf tissue. Phytochem. Bull..

[87] Sameeullah M., Sasaki T., Yamamoto Y. (2013). Sucrose transporter NtSUT1 confers aluminum tolerance on cultured cells of tobacco (Nicotiana tabacum L.). Soil Sci. Plant Nutr..

[88] Fuentes P., Zhou F., Erban A., Karcher D., Kopka J., Bock R. (2016). A new synthetic biology approach allows transfer of an entire metabolic pathway from a medicinal plant to a biomass crop. eLife.

[89] Mahmood K., Kannangara R., Jørgensen K., Fuglsang A.T. (2014). Analysis of peptide PSY1 responding transcripts in the two Arabidopsis plant lines: Wild type and psy1r receptor mutant. BMC Genom..

[90] Rosales-Mendoza S., Monreal-Escalante E., González-Ortega O., Hernández M., Fragoso G., Garate T., Sciutto E. (2018). Transplastomic plants yield a multicomponent vaccine against cysticercosis. J. Biotechnol..

[91] Latif S., Gottschamel J., Syed T., Younus I., Gull K., Sameeullah M., Batool N., Lössl A.G., Mariz F., Müller M. (2021). Inducible expression of human papillomavirus-16 L1 capsomeres in the plastomes of Nicotiana tabacum: Transplastomic plants develop normal flowers and pollen. Biotechnol. Appl. Biochem..

[92] Bradford M.M. (1976). A rapid and sensitive method for the quantitation of microgram quantities of protein utilizing the principle of protein-dye binding. Analytical Biochem..

[93] Nakagawa T., Kurose T., Hino T., Tanaka K., Kawamukai M., Niwa Y., Toyooka K., Matsuoka K., Jinbo T., Kimura T. (2007). Development of series of gateway binary vectors, pGWBs, for realizing efficient construction of fusion genes for plant transformation. J. Biosci. Bioeng..

[94] Xu K., Huang X., Wu M., Wang Y., Chang Y., Liu K., Zhang J., Zhang Y., Zhang F., Yi L. (2014). A rapid, highly efficient and economical method of Agrobacterium-mediated in planta transient transformation in living onion epidermis. PLoS ONE.

[95] Kim H.K., Choi Y.H., Verpoorte R. (2010). NMR-based metabolomic analysis of plants. Nat. Protoc..

[96] Bayramoğlu Karşı M.B., Yenisoy-Karakaş S., Karakaş D. (2018). Investigation of washout and rainout processes in sequential rain samples. Atmos. Environ..

[97] Ziaf K., Loukehaich R., Gong P., Liu H., Han Q., Wang T., Li H., Ye Z. (2011). A Multiple Stress-Responsive Gene ERD15 from Solanum pennellii Confers Stress Tolerance in Tobacco. Plant Cell Physiol..

