# Supplementary material for: Plastidial Expression of 3β-Hydroxysteroid Dehydrogenase and Progesterone 5β-Reductase Genes Confer Enhanced Salt Tolerance in Tobacco
Source: Int J Mol Sci. 2021 Oct 29;22(21):11736. doi: 10.3390/ijms222111736 (PMC8584194; doi:10.3390/ijms222111736)
Supplement: Supplementary file 1 [file ijms-22-11736-s001.zip › ijms-1407108-supplementary.pdf]

Supplementary Information

Supplementary Table S1. List of primers used in this study

Gene name/ Region of interest	Primer name	Primer sequence	Comments	Reference
3βHSD	*3βHSD*-ORF	5'-GGGGACAAGTTTGTACAAAAAAGCAGGCTT AATGTCGTCAAAGCCAAGGTTGG-3' 5'-GGGGACCACTTTGTACAAGAAAGCTGGGTT CTAACGCACGACGGTGAAGC-3'	Plastid transformation	This study
3βHSD	*3βHSD*_F (forward primer)	5'-ACGTCAGAGATGAAAAACAA-3'	To confirm presence and integration of *3βHSD* gene in chloroplast genome via PCR	This study
P5βR1	*P5βR1*_F (forward primer)	5'-CCCATGATCCACCCTACA-3'	To confirm presence and integration of *P5βR1* gene in chloroplast genome via PCR	This study
P5βR2	*P5βR2*_F (forward primer)	5'-TTAGACAACCTAATTTCTATTACAATCTAGAAG-3')	To confirm presence and integration of *P5βR2* gene in chloroplast genome via PCR	This study
Downstream of *trn*R (inside chloroplast genome)	oli252 (reverse primer)	5'-AGACAGCGACGGGTTCTCTG-3'	To confirm presence and integration of *3βHSD* in chloroplast genome via PCR End-to-end PCR	Gottschamel et al. (2013)
Upstream of *trn*N (inside chloroplast	oli253 (forward primer)	5'-GATCCGAGCCATAGAATTTC-3'	To confirm presence and integration of *aad*A in chloroplast	Gottschamel et al. (2013)

genome)			genome via PCR<br>End-to-end PCR	
Located inside *aad*A gene	oli059<br>(reverse primer)	5'-TGCTGGCCGTACATTTGTACG-3'	To confirm presence and integration of *aad*A in chloroplast genome via PCR	Gottschamel et al. (2013)
3βHSD	*3βHSD*-F<br>*3βHSD*-R	5'-GCTTACACGGCTTCCAAACA-3'<br>5'- CCCTTCAAGTTAGCCCTGGA-3'	For gene expression via RT-qPCR	This study
Actin9	actin9-F<br>actin9-R	5'-CCTGAGGTCCTTTTCCAACCA-3'<br>5'-GGATTCCGGCAGCTTCCATT-3'	For gene expression via RT-qPCR	Fuentes et al. (2016)
3βHSD	3βHSD-F<br>3βHSD-R	5'-GGGGACAAGTTTGTACAAAAAAGCAGGCTTA*atgtcgtcaaagccaaggttgg*-3'<br>5'-GGGGACCACTTTGTACAAGAAAGCTGGGTT*acgcacgacggtgaagc*-3'	Sub cellular localization	This study

Supplementary Figures

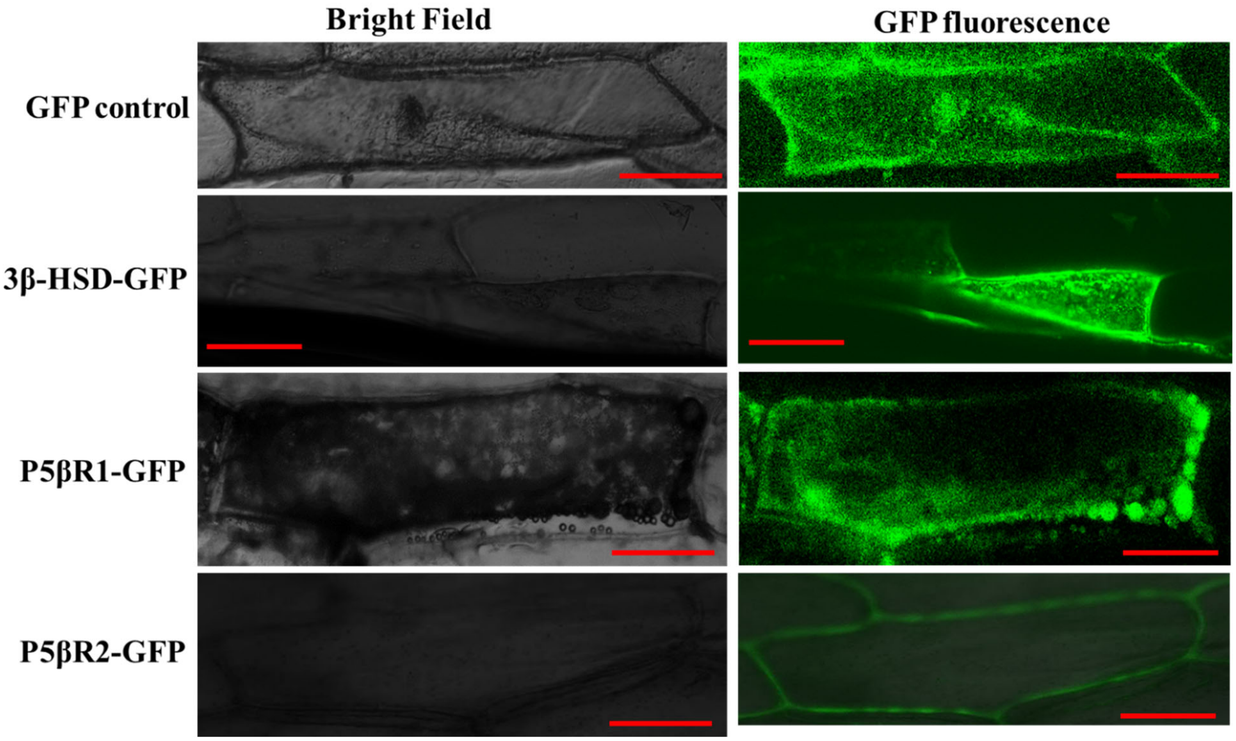

Supplementary Figure S1. Subcellular localization of *GFP* (upper panel) and *3β-HSD-GFP*, *P5βR1-GFP* and *P5βR2-GFP* (lower panel) transiently expressed in onion epidermal cells. Onion cells were transformed with the constructs for detection of subcellular localization of *3β-HSD-GFP*, *P5βR1-GFP*, *P5βR2-GFP* and GFP genes. Left, transmitted light images; Right, GFP fluorescence images; scale bar is 100 μm.

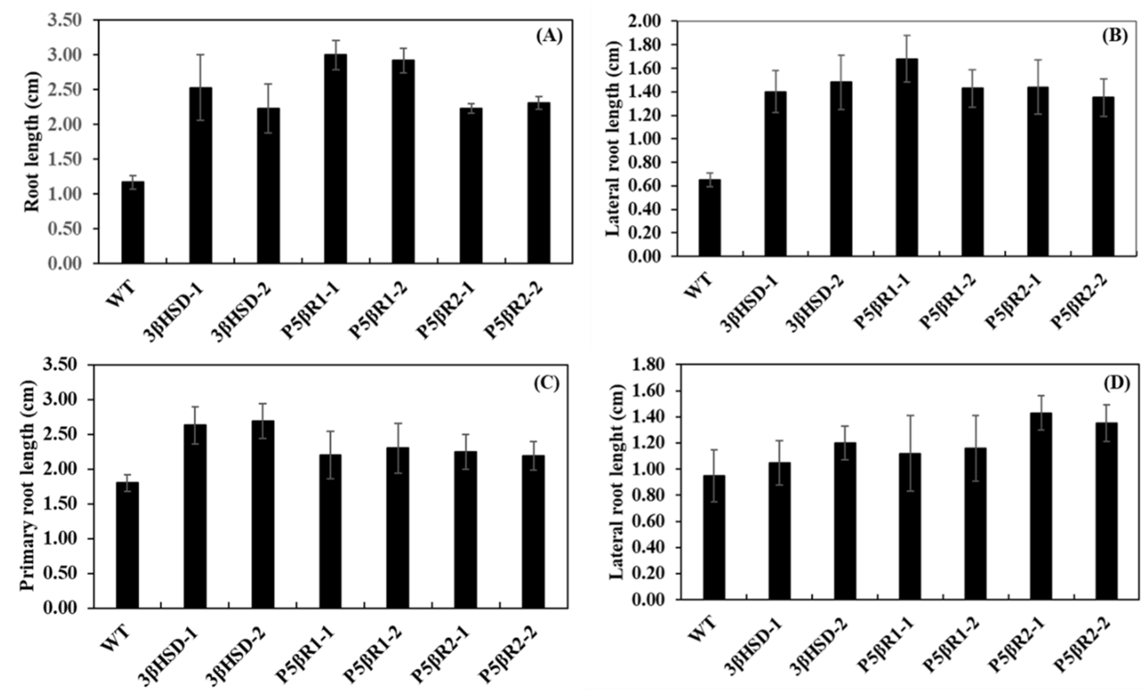

Supplementary Figure S2 Primary and lateral root lengths of WT and transplastomic lines of the *3β-HSD*, *P5βR1* and *P5βR2* genes under 50 mM and 200 mM NaCl concentrations on MS agar media over one month. (A, C) Primary root lengths of WT and two independent transgenic lines each of the *3β-HSD*, *P5βR1*, and *P5βR2* genes under 50 mM and 200 mM NaCl, (B, D) Lateral root lengths of the seedlings under 50 mM and 200 mM NaCl, (WT, n = 3, transplastomic lines, n=8 for each repeat of each line). Data shows average of three repeats and error bars represent ±SE.

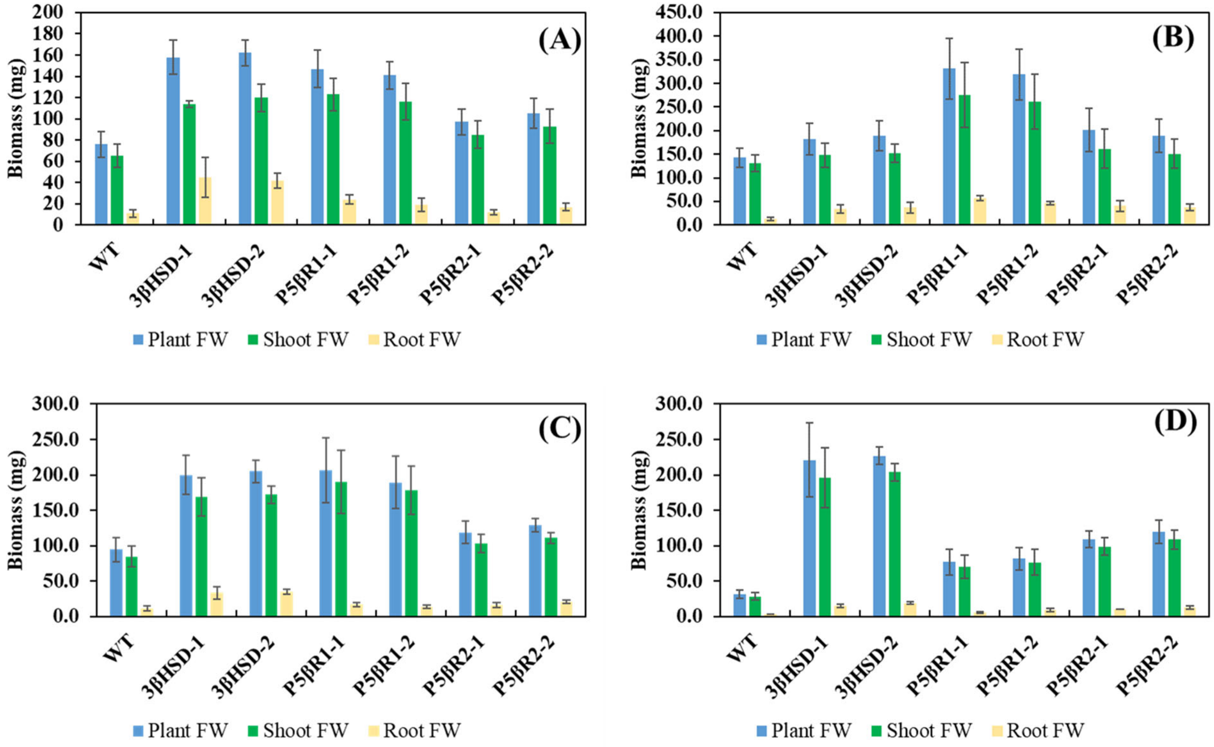

Supplementary Figure S3. Comparison of biomass of WT and transplastomic lines of the *3β-HSD*, *P5βR1* and *P5βR2* genes under control and various concentrations of NaCl stress. Fresh biomass of WT and two independent transgenic lines (3β-HSD-1, 3β-HSD-2, P5βR1-1, P5βR1-2, P5βR2-1 and P5βR2-2) of the *3β-HSD*, *P5βR1* and *P5βR2* genes. **(A)** Control treatment, **(B)** 50 mM NaCl, **(C)** 200 mM NaCl and **(D)** 300 mM NaCl. Data shows average of three repeats and error bars represent ±SE.

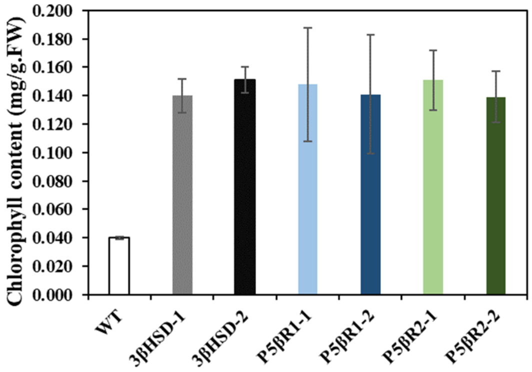

Supplementary Figure S4. Total chlorophyll content in WT and the independently generated transplastomic lines of 3β-HSD, P5βR1 and P5βR2 genes (3β-HSD-1, 3β-HSD-2, P5βR1-1, P5βR1-2, P5βR2-1 and P5βR2-2). Data shows average of three repeats and error bars represent ±SE.

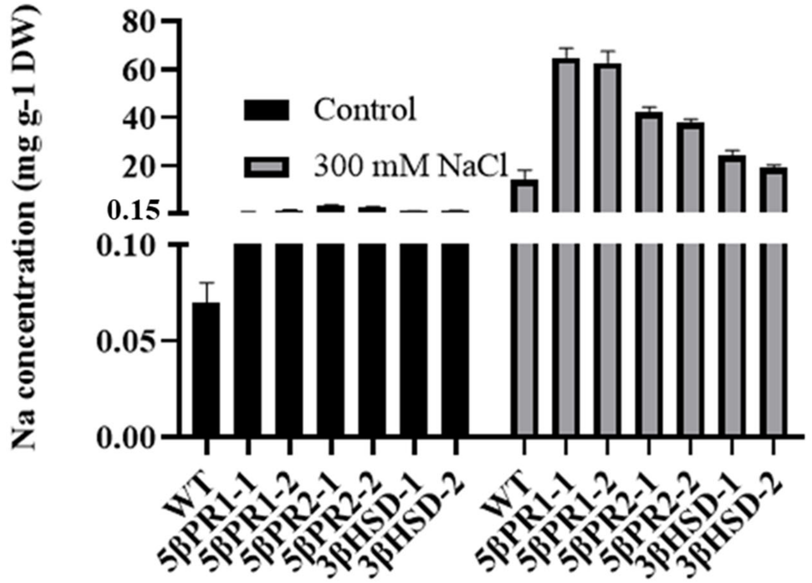

Supplementary Figure S5. **Level of Na concentration in the leaves of transplastomic and WT tobacco seedlings under control condition.** The Na^+ content in WT and the independently generated transplastomic lines of the 3β-HSD, P5βR1 and P5βR2 genes (P5βR1-1, P5βR1-2, P5βR2-1, P5βR2-2, 3β-HSD-1 and 3β-HSD-2) grown without NaCl stress. Data shows average of three repeats and error bars represent ±SE.

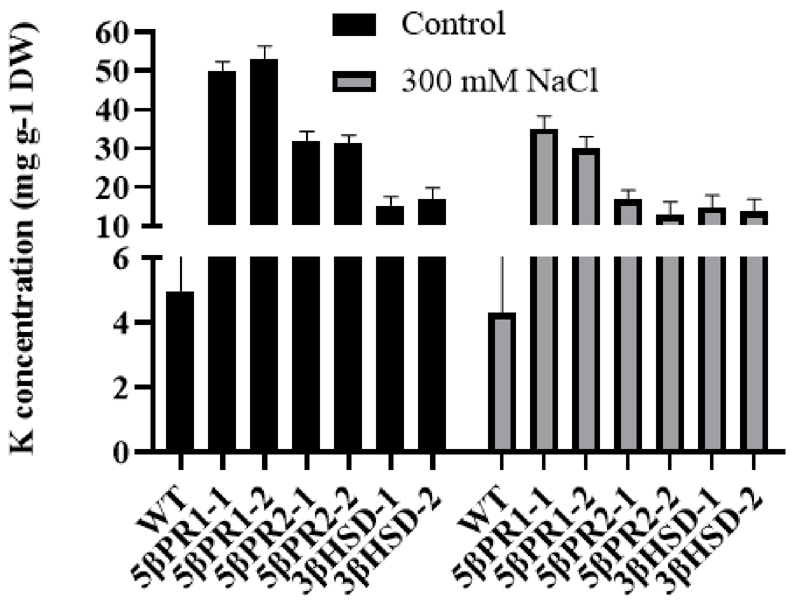

Supplementary Figure S6. **Level of K^+ concentration in the leaves of transplastomic and WT tobacco seedlings under control condition.** The K^+ content in WT and the independently generated transplastomic lines of the 3β-HSD, P5βR1 and P5βR2 genes (P5βR1-1, P5βR1-2,

P5βR2-1, P5βR2-2, 3β-HSD-1 and 3β-HSD-2) grown without NaCl stress. Data shows average of three repeats and error bars represent ±SE.
